# Differential neuronal functions of LNX1 and LNX2 revealed by behavioural analysis in single and double knockout mice

**DOI:** 10.1186/s12993-025-00276-z

**Published:** 2025-04-23

**Authors:** Laura Cioccarelli, Joan A. Lenihan, Leah G. Erwin, Paul W. Young

**Affiliations:** School of Biochemistry and Cell Biology, University College Cork, Cork, Ireland

**Keywords:** LNX1, LNX2, Anxiety, Risk-taking, Body weight, Ubiquitin ligase, PDZ domain, NUMB

## Abstract

**Background:**

Ligand of NUMB protein-X 1 (LNX1) and LNX2 proteins are closely related PDZ domain-containing E3 ubiquitin ligases that interact with and potentially modulate numerous synaptic and neurodevelopmentally important proteins. While both LNX1 and LNX2 are expressed in neurons, it is noteworthy that neuronal LNX1 isoforms lack the catalytic domain responsible for ubiquitination of substrates. Thus, the shared interaction partners of LNX1 and LNX2 might be differentially regulated by these proteins, with LNX1 acting as a stabilizing scaffold while LNX2 may promote their ubiquitination and degradation. Despite the identification of many LNX interacting proteins and substrates, our understanding of the distinct in vivo functions of LNX1 and LNX2 remains very incomplete.

**Results:**

We previously reported that mice lacking both LNX1 in the central nervous system and LNX2 globally exhibit decreased anxiety-related behaviour. Here we significantly extend this work by examining anxiety-related and risk-taking behaviours in *Lnx1*^*-/-*^ and *Lnx2*^*-/-*^ single knockout animals for the first time and by analysing previously unexplored aspects of behaviour in both single and double knockout animals. While the absence of both LNX1 and LNX2 contributes to the decreased anxiety-related behaviour of double knockout animals in the open field and elevated plus maze tests, the elimination of LNX2 plays a more prominent role in altered behaviour in the dark-light emergence test and wire beam bridge risk-taking paradigms. By contrast, *Lnx* knockout mice of all genotypes were indistinguishable from wildtype animals in the marble burying, stress-induced hyperthermia and novel object recognition tests. Analysis of the ultrasonic vocalizations of pups following maternal separation revealed significant differences in call properties and vocal repertoire for *Lnx1*^*-/-*^ and *Lnx1*^*-/-*^;*Lnx2*^*-/-*^ double knockout animals. Finally, decreased body weight previously noted in double knockout animals could be attributed largely to *Lnx1* gene knockout.

**Conclusions:**

These results identify specific roles of LNX1 and LNX2 proteins in modulating distinct aspects of anxiety and risk-taking behaviour and social communication in mice. They also reveal an unexpected role for neuronally expressed LNX1 isoforms in determining body weight. These novel insights into the differential neuronal functions of LNX1 and LNX2 proteins provide a foundation for mechanistic studies of these phenomena.

**Supplementary Information:**

The online version contains supplementary material available at 10.1186/s12993-025-00276-z.

## Introduction

The *L*igand of *N*UMB protein *X* (LNX) family of E3 ubiquitin ligases were named because of the ability of LNX1 and LNX2 to interact with NUMB – a negative regulator of Notch signalling during neurodevelopment and in other contexts [1, 2]. Both LNX1 and LNX2 can ubiquitinate specific isoforms of NUMB, targeting them for proteasomal degradation [3–5] and enhancement of Notch signalling by LNX1-mediated degradation of NUMB has been shown in cultured cells [4]. However, the influence of LNX proteins on Notch signalling in vivo in a mammalian context remains unclear.

The amino-terminal catalytic RING (*R*eally *I*nteresting *N*ew *G*ene) domain endows LNX1 and LNX2 with their ubiquitination activity, while four carboxyl-terminal PDZ (*P*SD-95, *D*lgA, *Z*O-1) domains can interact with numerous other proteins besides NUMB [6–9]. Some of these PDZ domain binding proteins are also substrates for LNX-mediated ubiquitination. It is noteworthy however that all LNX1 isoforms expressed in the central nervous system (CNS) lack intrinsic ubiquitination activity due to the absence of the RING domain [1, 10]. This points towards ubiquitination-independent neuronal functions of LNX1 involving its PDZ domains. By contrast only one, catalytically active, isoform of LNX2 exists and is expressed both peripherally and in the CNS [2]. This suggests that while many interaction partners are shared by LNX1 and LNX2, such proteins may be differentially regulated by LNX proteins in the CNS due to neuronal LNX1’s lack of ubiquitination activity.

Expression of *Lnx*1 and *Lnx2* mRNA is prominent in neurons in both the embryonic and adult CNS [1, 2]. LNX protein levels in the brain are very low however, with murine LNX1 only detectable following enrichment by immunoprecipitation [10, 11]. LNX2 is directly detectable by western blotting at late embryonic and early postnatal stages but its levels dramatically decrease thereafter [11]. These expression patterns are suggestive of a subtle role for LNX proteins in modulating brain development and/or activity.

To examine the in vivo functions of LNX proteins in the nervous system we previously generated double knockout mice lacking expression of the CNS-specific LNX1 isoforms (*Lnx1*^*exon3-/-*^ mice - hereafter referred to as *Lnx1*^*-/-*^) in addition to a global deletion of LNX2 [10, 11]. These *Lnx1*^*-/-*^;*Lnx2*^*-/-*^ double knockout mice are viable, healthy, fertile and exhibit normal motor and sensory function, though they do show a small but significant decrease in overall body weight. No gross neuroanatomical abnormalities were observed, arguing against a role for LNX proteins as major regulators of Numb/Notch signalling during brain development. In agreement with this, levels of NUMB protein in whole brain lysates were unaltered. The main behavioural alteration observed in these *Lnx1*^*-/-*^;*Lnx2*^*-/-*^ double knockout animals was decreased anxiety-related behavior in the open field and elevated plus maze paradigms [11].

Separately, Liu et al. [12] have reported deficits in social memory, decreased sociality and increased social avoidance in a different *Lnx1* knockout mouse line that is predicted to lack both the neuronal and non-neuronal isoforms of LNX1 protein. These deficits were attributed to a loss of LNX1 in the hippocampal CA3 region, where its mRNA expression is relatively high. Stabilization of postsynaptic EphB receptors in CA3 cells by LNX1 was previously shown to promote retrograde signalling that is required for proper mossy fibre axon targeting to CA3 and normal synaptic maturation [13]. In addition, reduced neuronal activity in the CA3 region during social interactions and a reduced ratio of NMDA: AMPA type glutamate receptors at mossy fibre-CA3 synapses was observed in *Lnx1*^-/-^ mice. Thus, at a molecular level, the disruption of a LNX1-GluN2B-EphB2 ternary complex in CA3 was proposed to underlie the altered social behaviour observed in these *Lnx1* null mice [12].

Here, building upon these previous studies, we performed a detailed analysis of stress, anxiety, risk taking and ultrasonic vocalisations in *Lnx1*^*-/-*^ and *Lnx2*^*-/-*^ single and double knockout animals of both sexes. The decreased anxiety-related behaviour seen for double knockout animals in the open field and elevated plus maze tests was reproduced and it was found that the absence of both *Lnx1* and *Lnx2* is required to observe a robust phenotype in these tests. Decreased anxiety-like or increased risk taking in the dark-light emergence test and wire beam bridge tests was observed for both double knockout and *Lnx2* single knockout animals, suggesting a more prominent role for LNX2 in these behavioural paradigms. Analysis of the ultrasonic vocalisations of pups following maternal separation showed increases in call length, power and delta frequency and a decrease in the principal frequency of calls for female *Lnx1*^*-/-*^;*Lnx2*^*-/-*^ mice, while call classification revealed differences in the vocal repertoire of both male and females *Lnx1*^*-/-*^ and *Lnx1*^*-/-*^;*Lnx2*^*-/-*^ animals. A persistent decrease in body weight compared to wild type animals was observed during postnatal development for *Lnx1*^*-/-*^ and *Lnx1*^*-/-*^;*Lnx2*^*-/-*^ but not *Lnx2*^*-/-*^ animals – indicative of a largely *Lnx1*-specific role. Overall, these observations pinpoint the specific roles of LNX1 and LNX2 proteins in modulating certain aspects of anxiety and risk-taking behaviour and social communication in mice.

## Methods

### Animals

*Lnx1*^*-/-*^ and *Lnx2*^*-/-*^ mice were obtained as previously described [11]. In *Lnx*^*-/-*^ mice, exon 3 - the first exon of the transcripts that codes for the p70 and p62 neuronal isoforms of LNX1, is replaced by a neomycin resistance gene abolishing transcription of these neuronal isoforms, but should not affect the expression of the non-neuronal LNX1 p80 isoform that is transcribed from a different upstream promoter [10]. *Lnx2*^*-/-*^ mice have a deletion of exon 2 which contains the ATG start codon and the coding region for the RING domain. The absence of LNX proteins in the CNS of these knockout lines has been previously verified [10]. Genotyping was performed on ear or toe biopsies taken for identification purposes as previously described [11]. *Lnx1*^*-/-*^ and *Lnx2*^*-/-*^ mice, which had previously been back crossed extensively to C57BL/6J mice were back crossed for two further generations before being crossed to generate double knockout *Lnx1*^*-/-*^;*Lnx2*^*-/-*^ mice. The genetic background of the knockout strains was checked through MiniMUGA genetic monitoring (Transnetyx, Cordova, TN, USA) [14]. For four DKO animals 98.4% of positions were called as C57BL/6JOlaHsd from 1926 total markers. This verified that the *Lnx* knockout lines are congenic on a C57BL/6JOlaHsd primary genetic background with a minor 129 secondary background. All animal experiments were approved by the Animal Experimentation Ethics Committee of University College Cork (No: 2021/029) and were conducted under license (No: AE19130/P168) issued by the Health Products Regulatory Authority of Ireland, in accordance with the European Union Directive 2010/63/EU for animals used for scientific purposes.

### Behavioural characterisation of mice

**Fig. 1:**
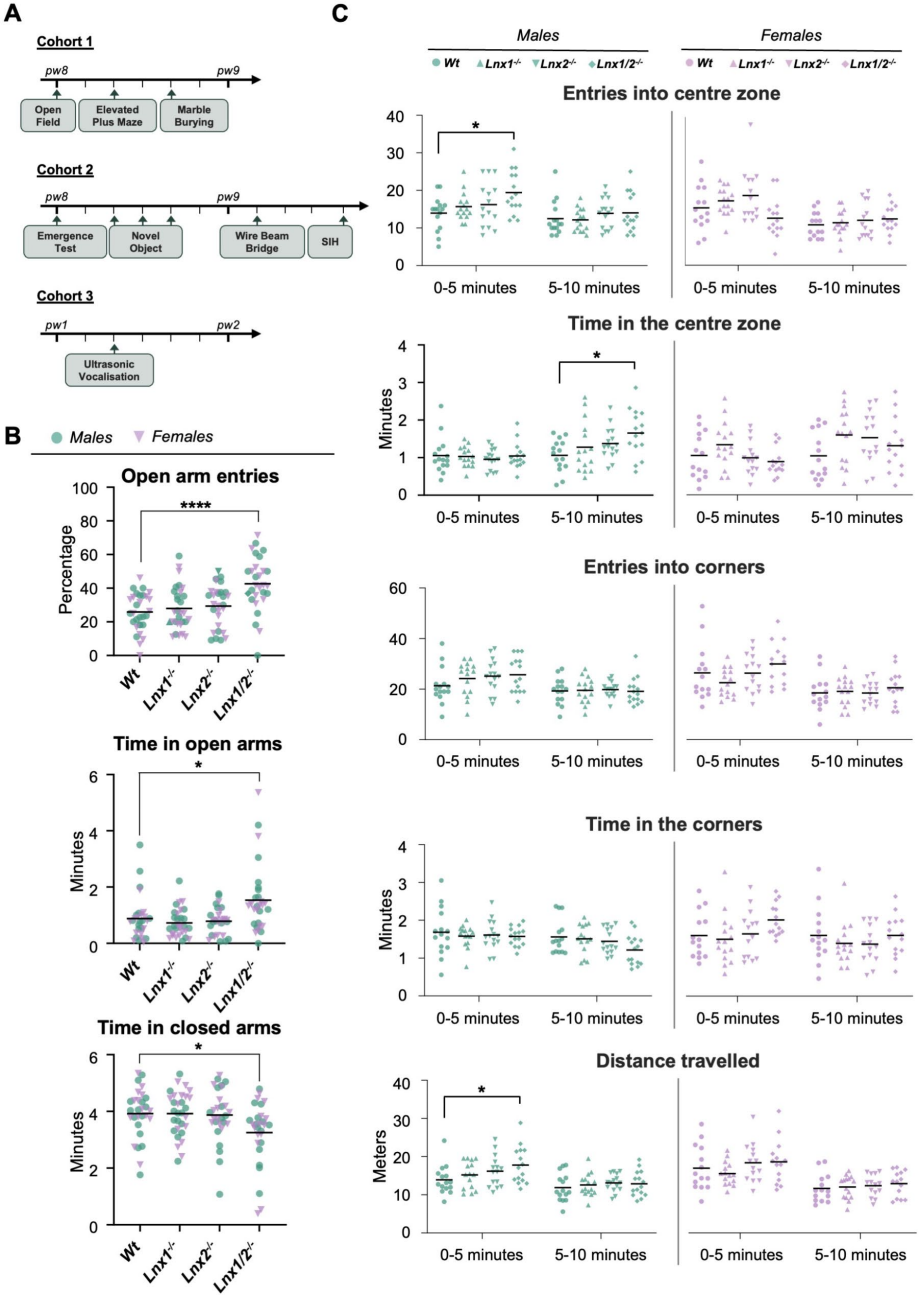
Effects of *Lnx* genotype on anxiety-related behaviour in the elevated plus maze and open field arena. **(A)** Schematic diagram illustrating the three cohorts of mice used for behavioural analysis and the timeline of testing. The number of days between each test is indicated. Each cohort comprised of ca. 15 male and 15 female mice of each genotype. pw = postnatal week, SIH = stress induced hyperthermia. **(B)** Effect of *Lnx* genotype on anxiety-related behaviour in the elevated plus maze. Mice of each genotype were tested for 6 min on the elevated plus maze and the indicated parameters calculated. Increased entries into, and time spent in the open versus the closed arms are indicative of reduced anxiety-like behaviour. *n* = 29–30 per group; Statistical significance determined by Kruskal-Wallis / Dunn’s multiple comparisons tests. **(C)** Effect of *Lnx* genotype on spontaneous locomotor activity and anxiety-related behaviour in the open field arena. Mice of the indicated genotypes were placed in the centre of the arena and allowed to move freely for 10 min (divided into two 5-minute bins for analysis). Distance travelled was analysed as an index of general locomotor activity. The number of entries into, and amount of time spent in the centre versus the corners of the arena were monitored as indicators of differences in anxiety-like behaviour. *n* = 14–15 per group. Statistical significance determined by Kruskal-Wallis / Dunn’s multiple comparisons tests for time in centre, time in corners and entries into centre, and by ANOVA / Dunnett’s multiple comparisons test for entries into corners and distance travelled. *p < 0.05, **p < 0.01, ***p < 0.001

Separate colonies of wild type (*Wt*), *Lnx1*^*−/−*^, *Lnx2*^*−/−*^ and *Lnx1*^*−/−*^;*Lnx2*^*−/−*^ double knockout mice were established and bred to obtain all mice required for this study. Males were removed and females singly housed before parturition. At weaning (P21), mice were separated by sex and housed as mixed genotypes, 2–5 mice per individually ventilated cage. Cages were environmentally enriched, and mice had *ad libitum* access to food and water. Mice were maintained on a 12 h light/dark cycle (lights on at 07:30), under temperature (22 ± 1 ºC) and humidity-controlled conditions. Behavioural testing was performed on three separate cohorts of mice (Fig. 1A). Typically, each cohort of mice consisted of 15 animals of each sex for each genotype. Exact numbers ranged from 12 to 17 mice and are provided for each test in Additional File 1. No more than three mice of a given sex from the same litter were included in a cohort. There was a minimum rest period of at least 24 h between each test. Animals were habituated to the test room at least 30 min prior to testing, unless stated otherwise. The researcher left the room after the start of all trials that were video recorded. All experiments were conducted during the light phase of the day. All apparatus were cleaned between animals with 70% ethanol to remove odours. Genotypes were blinded for the duration of the behavioural testing, and for subsequent scoring.

### Elevated plus maze

The elevated plus maze (Stoelting Co., IL, USA) consisted of four arms (5 cm wide x 35 cm long) radiating from a centre platform area (5 × 5 cm) in a plus formation, that was elevated 50 cm above the floor. Two opposing arms were enclosed by 15 cm high walls, the other two arms were open. The experiment was performed under dim red light (5 lx at the centre of the maze). Mice were individually placed on the centre platform of the maze, facing an open arm, and allowed to freely explore the maze for 6 min. As indices of anxiety-like behaviour, time spent in the open and closed arms of the maze were measured post-test using a video-tracking system (Any-maze software 7.3, Stoelting Co., IL, USA), while the number of entries into the open and closed arms were scored manually (with the animal adjudged to have entered an arm of the maze only when all four paws were inside the arm in question).

### Open field

The apparatus consisted of a grey, plastic, open box (45 × 45 × 40 cm, L × W × H), without any bedding. Light levels were adjusted to an intensity of 60 lx in the centre of the arena. After 60 min habituation to the testing room, animals were placed individually in the middle of the arena and allowed 10 min of free exploration. Total distance travelled, time spent and number of entries into the centre and the four corner areas of the arena were measured post-test using a video-tracking system (Any-maze software 7.3, Stoelting Co., IL, USA), where the centre area was defined as a 27 × 27 cm square in the middle of the arena (36% of total arena) and the corners were delineated as 9 × 9 cm squares.

### Dark–light emergence test

Mice were individually placed in a plastic, opaque shelter (11 × 7 × 7 cm, L x W x H) positioned centrally along one wall of a brightly illuminated (circa 600 lx) open field arena (described above). The shelter contained a small opening (4 × 4 cm) with a sliding door, orientated towards the centre of the arena, that remained closed for an initial 5-minute habituation period. The door was then opened, and animal movements were recorded for 5 min. Latency to emerge from the shelter, time spent in the open arena, and number of transitions to and from the enclosure during this period were scored manually post-test. Emergence and transitions were counted only when all four paws had crossed into the area in question.

### Wire beam Bridge test

The apparatus, elevated 60 cm above the floor, consisted of a black, open-front enclosure (14 × 14 × 14 cm, L x W x H), connected to a circular platform (6 cm diameter, containing 20 g regular food pellets), by an unrailed bridge (30 cm long, 1.5 cm wide). The bridge was constructed from plastic coated gardening wire and was somewhat flexible, with a downward deflection of ~ 1 cm per 100-g load at the centre point. It consisted of two parallel beams (wire thickness: 2 mm) perpendicularly connected by 24 equally distanced cross-ties (wire thickness: 1.2 mm). Black shields were placed around the apparatus to minimise potential distractions from the surrounding environment. The test was conducted under dim light (10 lx at centre of bridge). Following 5 min habituation to the elevated enclosure, mice were briefly returned to their home cage, while the bridge and platform were positioned. Mice were then returned to the enclosure and allowed to freely explore the apparatus for 10 min. Latencies to access the bridge (all four paws on the bridge) and the food (entire head reaching the platform) were scored manually post-test. Animals that fell during the habituation period were excluded from the analysis.

### Marble burying

Mice were individually placed in a cage (38 × 25 × 18 cm, L x W x H, circa 250 lx), prefilled with clean corncob bedding to a depth of 5 cm, for a 20 min habituation period. Subsequently, mice were briefly removed, while 20 clean, black, glass marbles (1.4 cm diameter) were overlaid on the bedding, equidistant from each other, in a 4 × 5 arrangement. Mice were then returned to the experimental cage and were left undisturbed for a further 30 min. The number of marbles that had two thirds or more of their surface covered by bedding at the end of the test period were counted independently by two researchers, and averaged. Video recording was not performed for this test and thus the possibility of an animal repeatedly reburying the same marble multiple times was not assessed.

### Stress induced hyperthermia

Mice that had been singly housed for 48 h were brought to the test room individually, immediately prior to testing. Mice were hand-restrained and held horizontally, while a prelubricated temperature probe (TME 2000, Single Input Thermocouple Thermometer) was gently inserted into the rectum, to a fixed depth of 2 cm. Rectal temperature was measured in this way twice with a 15-min interval, where the mice were returned to their home cage. The increase in temperature from the first to second measurement (due to the stress experienced during the first temperature measurement) was calculated as the stress-induced hyperthermia response.

### Novel object recognition test

The test involved three trials of 10 min each, separated by an inter-trial interval of 24 h performed in the open field arena described above with the light level set to approx. 15 lx. In trial one the test mouse was allowed to freely explore the empty arena for acclimatization. In the second trial two of the same objects were placed approximately 10 cm from either corner of the arena and the mouse was placed facing the opposite wall to start the trial. In the third trial one of the previously used objects was replaced by a different object and the trial repeated as before. The objects used were a flat bottomed, transparent rectangular Sarstedt T-75 tissue culture flask (8 × 4 × 13 cm, L x W x H) partially filled with blue dye and a grey ribbed opaque cup with a light blue lid (6 cm & 9 cm in diameter (base & top), 13 cm high). Objects were affixed to the floor of the arena. The lack of any intrinsic preference for either of the objects was tested in a pilot study; the use of objects as the familiar versus novel object and the position of the objects (left vs. right) was randomized for each test mouse. The exploration time for novel and familiar objects was scored manually from video recordings. Pointing and sniffing the object in an area of 2 cm around it, as well as touching and climbing the object were counted as interaction; grooming in the interaction zone or staying on the top of the object was not. Total exploration time on day 2 was used as an indicator of general novelty-seeking behaviour. Individuals exploring each object for less than 20s on day 2 were excluded from the analysis of day 3. To analyse differences between groups a ‘Discrimination Index’ ((novel object exploration time / total object exploration time) x 100) was used.

### Recording and analysis of ultrasonic vocalisations

Ultrasonic vocalisations were recorded immediately following maternal separation in males and females at postnatal day 9. Each pup was gently removed from their mother and littermates and placed into a plastic open-top isolation chamber (11.5 × 7.5 × 4 cm), without bedding material. The isolation chamber was then covered with a sound-attenuating Styrofoam box with a microphone (Petterson M500, USB Ultrasound Microphone) suspended from the roof, 4 cm above the pup. The emitted vocalisations were recorded over a 3-minute period using UltraVox XT 3.2 software (Noldus Information Technology). Recordings were analysed using DeepSqueak, a deep learning-based vocalization detection and analysis tool (Coffey et al., 2019) via MATLAB R2024a (The MathWorks Inc., Natick, MA, USA). The minimum and the maximum cutoff frequencies were set at 30 kHz and 150 kHz. Spectrotemporal contours were extracted using the default threshold settings. Calls identified as noise were manually excluded. Number of USVs was calculated for all pups. Individuals with < 3 USVs were then excluded from the analysis to ensure that reliable average values could be calculated for the following parameters: latency to the first call, the average length of USVs (s), the principal frequency (median frequency of the contour(kHz)), the delta frequency (highest – lowest frequency of the contour (kHz)) and the mean power (dB/Hz). Three animals in total, each of a different genotype, were excluded based on having < 3 USVs. Vocalisations were then classified using the supervised neural network-based classification available in DeepSqueak. To train the algorithm, one individual from each genotype and sex was randomly selected (8 in total) and their USVs were manually classified into 7 different categories: Up, Down, Short, Chevron, Complex, Multi Simple and Multi Complex. The classification of vocalisation was adapted from Scattoni et al. [15] and modified as follows: multi-component calls containing only simple call components (Up, Down, Short and Chevron) were classified as *Multi Simple*, multi-component calls containing at least one complex call component were classified as *Multi Complex*.

### Statistical analysis

Statistical analyses were performed using GraphPad Prism v.10.2.3 (Boston, MA, USA). The normal distribution of behavioural data was assessed using the Shapiro-Wilk test. In line with local ethical guidance on reducing numbers of animals used for experimentation, results from males and females were combined for analysis when sex was determined not to be a factor affecting any of the dependent variables measured in a given test. To stringently test for effects of sex in each behavioural paradigm, data from males and females for each parameter of interest were compared for each genotype using t-tests or Mann Whitney U-tests (for normally and non-normally distributed data respectively; see Additional File 1). If no significant effects of sex (*P* < 0.05) were observed for any parameter/genotype combination then subsequent analysis was performed on combined data from both sexes, otherwise data from males and females was analysed separately. In most cases data from LNX knockout genotypes was compared to that of wildtype animals using one-way ANOVA followed by Dunnett’s post hoc test or Kruskal-Wallis analysis followed by Dunn’s multiple comparisons test (for normally and non-normally distributed data respectively). Body weight data was analysed using a two-way ANOVA with repeated measures performed on rank transformed data followed by a Dunnett’s multiple comparisons test. *P* values of less than 0.05 were considered significant and differences are indicated in the figures by *P ≤ 0.05, **P ≤ 0.01 and ***P ≤ 0.001. A summary of all statistical analyses to examine effects of *Lnx* genotype is provided in Additional File 2. Results of most behavioural experiments are presented as scatter plots in which single dots/lines, colour-coded by sex, represent individual mice (to indicate the distribution of the data) and mean values are shown by a thick black line.

## Results

### Loss of both LNX1 and LNX2 contribute to decreased anxiety-like behaviour of Lnx1^*−/−*^;*Lnx2*^*−/−*^ mice in the elevated plus maze

We previously observed decreased anxiety-related behaviour in *Lnx1*^*-/-*^;*Lnx2*^*-/-*^ double knockout mice in the elevated plus maze test [11], which is based on rodents’ preference to explore and spend time in the “safer” environment of the closed, versus the open arms of the maze [16]. To determine whether knockout of either the *Lnx1* or *Lnx2* gene alone was sufficient to cause this phenotype *Wt*, *Lnx1*^*-/-*^, *Lnx2*^*-/-*^ and *Lnx1*^*-/-*^;*Lnx2*^*-/-*^ mice were examined in this paradigm. Since no significant differences between males and females were observed for any of the assessed parameters (Additional File 1), the sexes were combined for subsequent analyses. A significant effect of genotype was detected for the percentage of entries into (H (3) = 22.16, *p = <* 0.0001), and the time spent in the open arms (H (3) = 13.64, *p* = 0.0034; Kruskal-Wallis; Fig. 1B). Dunn’s multiple comparisons test revealed that *Lnx1*^*-/-*^;*Lnx2*^*-/-*^, but not *Lnx1*^*-/-*^ or *Lnx2*^*-/-*^ mice, entered the open arms more frequently (*p = <* 0.0001) and spent significantly more time in the open arms (*p* = 0.0141) compared to *Wt* counterparts. Conversely, a significant effect of genotype on time spent in the closed arms (H (3) = 10.19, *p =* 0.017) was also observed, with *Lnx1*^*-/-*^;*Lnx2*^*-/-*^(*p* = 0.0159), but not *Lnx1*^*-/-*^ or *Lnx2*^*-/-*^ mice spending significantly less time in the closed arms compared to WT animals. These findings recapitulate the reduced anxiety-like behaviour previously seen for *Lnx1*^*-/-*^;*Lnx2*^*-/-*^ mice in this test [11] and demonstrate that elimination of both LNX1 and LNX2 expression is required to robustly observe this phenotype.

Spontaneous locomotor activity was assessed in the open field arena over a 10-minute trial with results analysed in two 5-minute bins. In this test time spent in, and entries into, the corners versus the exposed centre of the arena were considered as measures of anxiety-like behaviour (Crawley, 2008). Total distance travelled served as an index of locomotor activity. Significant effects of sex were observed for several parameters in this test (Additional File 1) and thus males and females were analysed separately. For male mice (Fig. 1C, left panels), a near significant effect of genotype on number of entries into the centre of the arena in the first half of the trial (H (3) = 6.351, *p =* 0.0957) and time spent in the centre of the arena in the second half of the trial (H (3) = 7.497, *p =* 0.0576; Kruskal-Wallis) were apparent. Dunn’s multiple comparisons test revealed significant differences between *Lnx1*^*−/−*^;*Lnx2*^*−/−*^ and *Wt* mice in both cases (*p* = 0.0457 and *p* = 0.0295 respectively). A reciprocal trend was seen for time spent in the corners of the arena, though without reaching statistical significance. Male *Lnx1*^*−/−*^;*Lnx2*^*−/−*^ animals also travelled a greater distance than *Wt* mice in the first half of the trial (F_3,56_ = 2.355, *p* = 0.0817; One-way ANOVA and *p* = 0.0338; Dunnett’s test). These findings indicate that *Lnx1*^*−/−*^;*Lnx2*^*−/−*^ male mice show greater initial exploration of and then spend more time in the exposed centre of the arena, consistent with previous observations of decreased anxiety-like behaviour in the open field [11]. By contrast, for females, no significant differences between *Wt* and *Lnx* knockout mice of any genotype were discernible for any parameters in the current analysis (Fig. 1C, right panels).

### Prominent role for LNX2 knockout in anxiolytic and enhanced risk-taking in the dark-light emergence and wire beam bridge tests

**Fig. 2:**
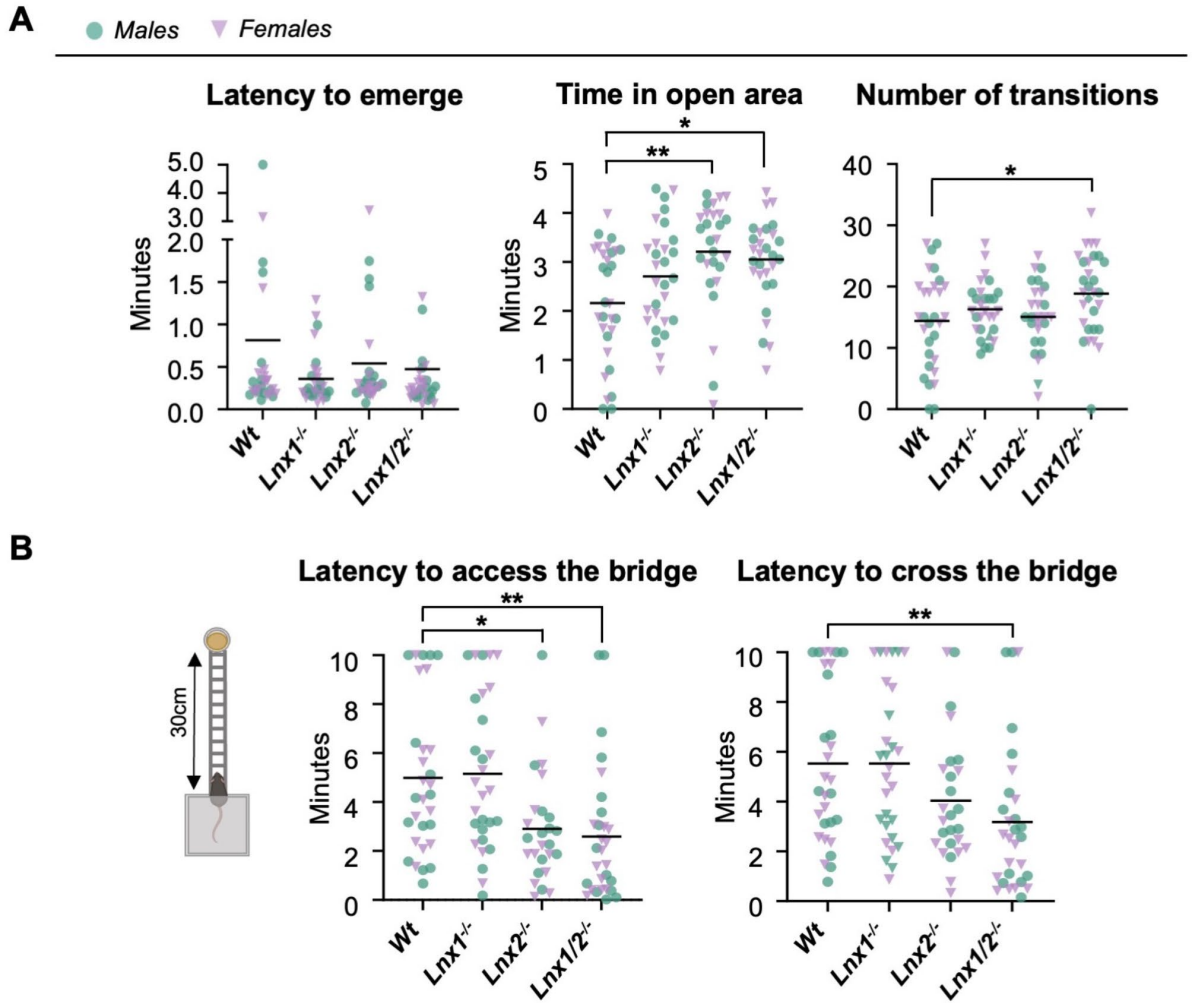
Effects of *Lnx* genotype on anxiety-related and risk-taking behaviour in the dark-light emergence and wire beam bridge tests. **(A)** Effect of *Lnx* genotype on anxiety-related behaviour in the emergence test. Mice of each genotype were released from a small covered enclosure placed within an open field arena. Latency to emerge from the enclosure, time spent in the open area and number of transitions to and from the enclosure were monitored as indicators of risk-taking and anxiety-related behaviour. *n* = 27–30 per group; Statistical significance determined by Kruskal-Wallis / Dunn’s multiple comparisons tests for time in the open area and latency to emerge, and by ANOVA / Dunnett’s multiple comparisons tests for number of transitions. **(B)** Effect of *Lnx* genotype on risk-taking behaviour in the wire beam bridge test. Mice of each genotype were tested for 10 min on the apparatus and time taken for the mice to first step onto the bridge with all four paws and to first reach the platform with food were calculated. Smaller latency values for these parameters are indicative of reduced anxiety-like or increased risk-taking behaviour. *n* = 26–30 per group; Statistical significance determined by Kruskal-Wallis / Dunn’s multiple comparisons tests. *p < 0.05, **p < 0.01, ***p < 0.001

Next, to capture distinct aspects of anxiety-related behaviour, *Lnx* single and double knockout mice were examined in some additional tests that had not been employed in our previous analysis. We used the dark-light emergence test paradigm to examine the conflict between spontaneous exploratory behaviour and the innate aversion of mice to brightly illuminated areas [17, 18]. Males and females were analysed together since no significant between-sex differences were observed (Additional File 1). No difference between *Wt* and *Lnx* knockout genotypes was detected for latency to first enter the open, illuminated area from the covered enclosure (H (3) = 3.370, *p* = 0.3380; Kruskal-Wallis; Fig. 2A). However, a significant effect of genotype was detected for time spent in the open area (H (3) = 13.97, *p =* 0.0029; Kruskal-Wallis) and number of transitions to and from the enclosure (F_3,112_ = 2.897, *p* = 0.0383; One-way ANOVA). Post-hoc analysis revealed that both *Lnx2*^*-/-*^ and *Lnx1*^*-/-*^;*Lnx2*^*-/-*^ mice spent significantly more time in the open area compared to *Wt* animals (*p* = 0.0013 and *p* = 0.0258; Dunn’s test), while *Lnx1*^*-/-*^;*Lnx2*^*-/-*^ mice made significantly more transitions to and from the enclosure (*p* = 0.0194; Dunnett’s test).

The wire beam bridge test has been used to assess aspects of impulsivity, sensation-seeking, and risk-taking behaviours in mice [19, 20] (Fig. 2B). In this paradigm latencies to access and cross the bridge were scored as indicators of exploratory risk-taking behaviour. Males and females were analysed together since no significant between-sex differences were observed (Additional File 1). Significant effects of genotype on latency to access and to cross the bridge were observed (H (3) = 19.94, *p =* 0.0002 and H (3) = 13.60, *p =* 0.0035 respectively; Kruskal-Wallis). Post-hoc analysis revealed that both *Lnx2*^*−/−*^ and *Lnx1*^*−/−*^;*Lnx2*^*−/−*^ mice took significantly less time to initially access the bridge (*p* = 0.0266 and *p* = 0.0018; Dunn’s test) compared to *Wt* animals, while only *Lnx1*^*−/−*^;*Lnx2*^*−/−*^ mice took significantly less time to cross the bridge and reach the platform with food pellets (*p* = 0.0058; Dunn’s test). Overall, the results of the dark-light emergence and wire beam bridge tests clearly demonstrate decreased anxiety-related or increased risk-taking behaviour in *Lnx1*^*−/−*^;*Lnx2*^*−/−*^ mice and suggest a more prominent involvement of *Lnx2* versus *Lnx1* knockout in causing this phenotype.

### Lnx knockout and Wt mice are indistinguishable in the marble burying, stress induced hyperthermia and novel object recognition tests

**Fig. 3:**
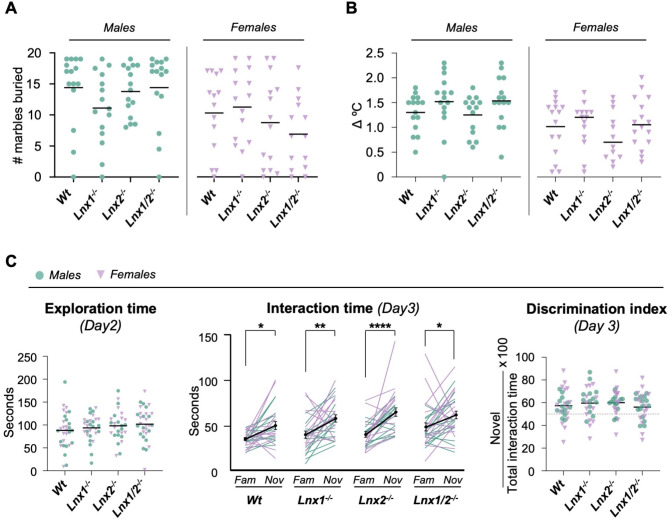
Analysis of Lnx knockout mice in the marble burying, stress induced hyperthermia and novel object recognition tests. **(A)** Effect of *Lnx* genotype on performance in the marble burying task. Mice of the indicated genotypes were placed in a cage with 20 marbles for 30 min. The number of marbles buried at the end of this time was quantified as a measure of obsessive-compulsive related anxiety-like behaviour. *n* = 14–15 per group. Statistical significance determined by Kruskal-Wallis / Dunn’s multiple comparisons tests for males and one-way ANOVA / Dunnett’s multiple comparisons tests for females. *p < 0.05, **p < 0.01, ***p < 0.001. **(B)** Responses of *Lnx* knockout and *Wt* mice assessed in the stress-induced hyperthermia paradigm. Change in body temperature (T_15min_ – T_0min_) is presented for males and females. *n* = 14–15 per group. Statistical significance determined by Kruskal-Wallis / Dunn’s multiple comparisons tests. **(C)** Object recognition memory assessed in the novel object recognition test. Total exploration of both objects in trial 2 of the task – quantified as an indicator of general novelty-seeking behaviour (left panel). Interaction time with each object during trial 3 of the task (middle panel). Statistical significance of differences in interaction time with the familiar versus novel objects for each genotype were determined by Kruskal-Wallis / Dunn’s multiple comparisons tests. Discrimination index to compare performance of mice of the indicated genotypes in trial 3 of the task (right panel). Statistical significance assessed by ANOVA / Dunnett’s multiple comparisons tests. *p < 0.05, **p < 0.01, ***p < 0.001

Marble burying has been used to quantify obsessive-compulsive-like anxiety behaviour, with the burying of marbles regarded as a compulsive behaviour to relieve anxiety in response to an anxiogenic stimulus [16, 21]. To explore this aspect of anxiety in *Lnx* knockout mice, the number of marbles buried in a 30 min trial by male and female mice of each genotype was quantified (Fig. 3A). In general, males buried more marbles than females and this difference was statistically significant for several genotypes (Additional File 1), therefore males and females were analysed separately. The number of buried marbles was not significantly different between genotypes for either males (H (3) = 4.755, *p =* 0.1907; Kruskal-Wallis) or females (F_3,53_ = 1.292, *p* = 0. 2867; one-way ANOVA) (Fig. 3A).

Given the altered behaviour of *Lnx1*^*−/−*^;*Lnx2*^*−/−*^ mice in mildly stressful situations such as the elevated plus maze or wire beam bridge test, stress response was examined more generally in our *Lnx* knockout lines. To this end stress induced hyperthermia, the autonomic increase in body temperature following exposure to stressful stimulus [22], was evaluated in *Wt*,* Lnx1*^*−/−*^, *Lnx2*^*−/−*^ and *Lnx1*^*−/−*^;*Lnx2*^*−/−*^ animals. The stress induced hyperthermia response was significantly different between males and females (Additional File 1), therefore comparisons between genotypes were performed separately for each sex. The stress induced hyperthermia response was not significantly different between genotypes for either males (H (3) = 5.477, *p =* 0.14; Kruskal-Wallis) or females (H (3) = 1.589, *p =* 0.6618; Kruskal-Wallis; Fig. 3B).

We previously found that short-term spatial working memory in *Lnx1*^*−/−*^;*Lnx2*^*−/−*^ as assessed by spontaneous alternation in a Y maze was normal [11]. To further test cognitive function and evaluate longer term object recognition memory we performed the novel object recognition test with a 24 h interval between trials [23]. Males and females were analysed together since no significant between-sex differences were observed (Additional File 1). On day two, when mice are being familiarized with two identical objects, total exploration of the objects was quantified as an indicator of general novelty-seeking behaviour [24], and no significant effects of genotype were observed (Fig. 3C, left panel). The following day, in trial 3 of the task, when mice were presented with one novel and one familiar object, wildtype as well as all *Lnx* knockout genotypes exhibited significantly more exploration of the novel compared to the familiar object (H (7) = 48.69, *p = <* 0.0001; Kruskal-Wallis; Fig. 3C, middle panel). To test for differences between genotypes a discrimination index that represents the ratio of time spent exploring the novel versus familiar object was calculated (Fig. 3C, right panel). No significant differences were observed for any of the *Lnx* knockout genotypes versus wildtype animals (F_3,105_ = 0.5474, *p* = 0.6510; one-way ANOVA). Overall, these results indicate that *Lnx* knockout mice of all genotypes have normal object recognition memory, normal changes in body temperature in response to stress and behave similarly to *Wt* animals in the marble burying task.

### Call characteristics and vocal repertoire of ultrasonic vocalisations are altered in Lnx1^−/−^ and Lnx1^−/−^;Lnx2^−/−^ pups

Ultrasonic vocalisations (USVs) in mice are a means of communication in various social contexts that may reflect internal emotional or motivational states [25, 26]. We reasoned that examining USVs in *Lnx* knockout pups separated from their mothers could provide insights into their anxiety and stress responses, as well as their social communication – the latter being of interest given the abnormalities in social behaviour previously reported in a *Lnx1* knockout line [12].

**Fig. 4:**
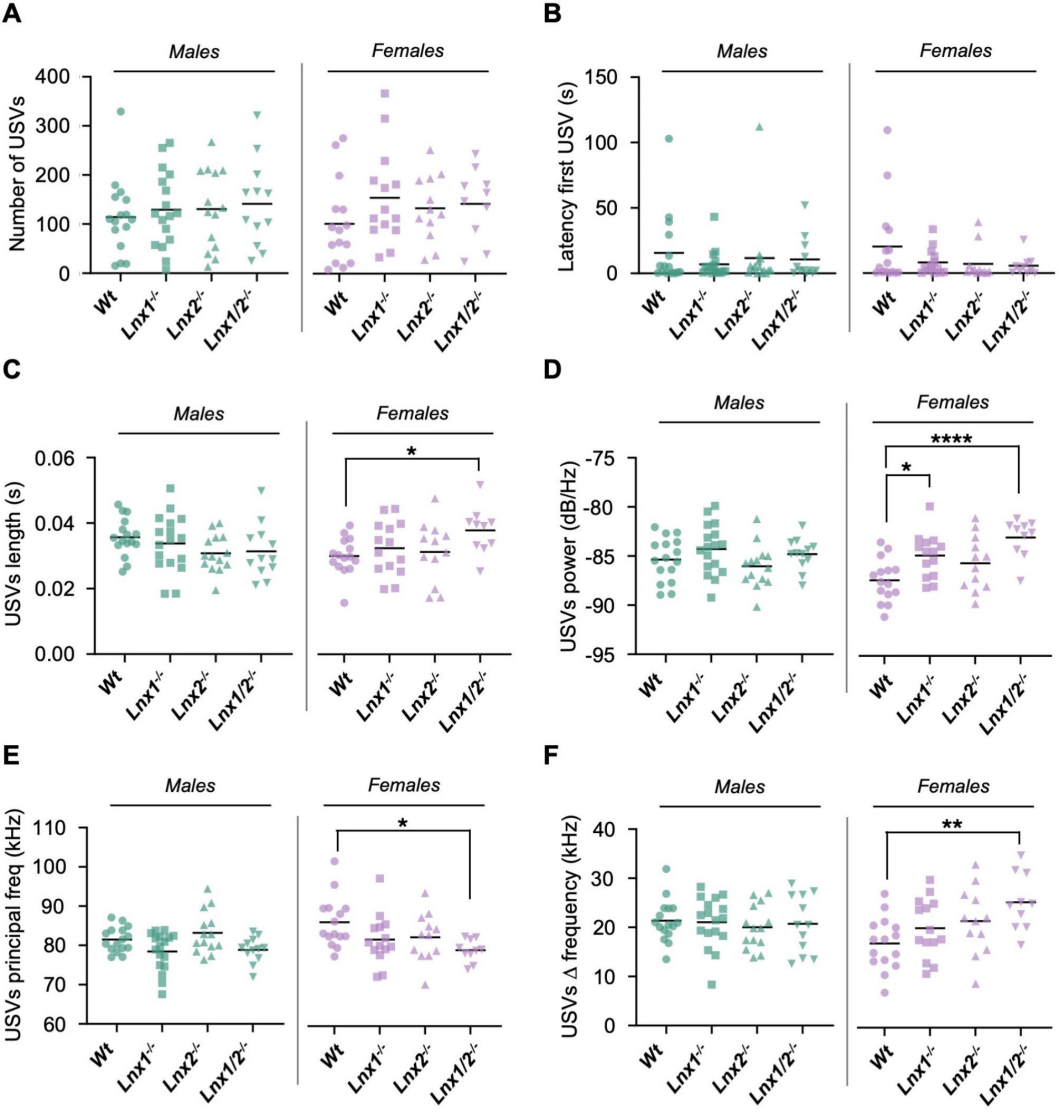
Basic USV call properties of *Lnx* knockout mice. **(A-F)** Quantification of the indicated features of USV calls recorded during 3 min of maternal separation presented as scatter plots of values for individual mice with the mean represented as a horizontal line. Statistical significance assessed by Kruskal-Wallis / Dunn’s multiple comparisons tests **(A, B)** or one way ANOVA / Dunnett’s multiple comparisons tests (C-F). *n* = 10–15 per group. *p < 0.05, **p < 0.01, ***p < 0.001

USVs in postnatal day 9 pups were recorded for 3 min upon separation from their mother. Significant effects of sex were observed for several parameters in this test (Additional File 1) and thus males and females were analysed separately. The total number of calls was not significantly different for any *Lnx* knockout genotype compared to *Wt* pups, nor was the latency to the first vocalisation (Fig. 4A-B). Characterisation of basic call properties did not reveal any differences based on genotype for males. However, for females, mean call length and delta frequency were significantly increased, while the principal frequency of calls was decreased for *Lnx1*^*−/−*^;*Lnx2*^*−/−*^ in comparison to *Wt* mice (Fig. 4C-E). In addition, the power of vocalisations was significantly higher for both *Lnx1*^*−/−*^ and *Lnx1*^*−/−*^;*Lnx2*^*−/−*^ mice (Fig. 4F).

**Fig. 5:**
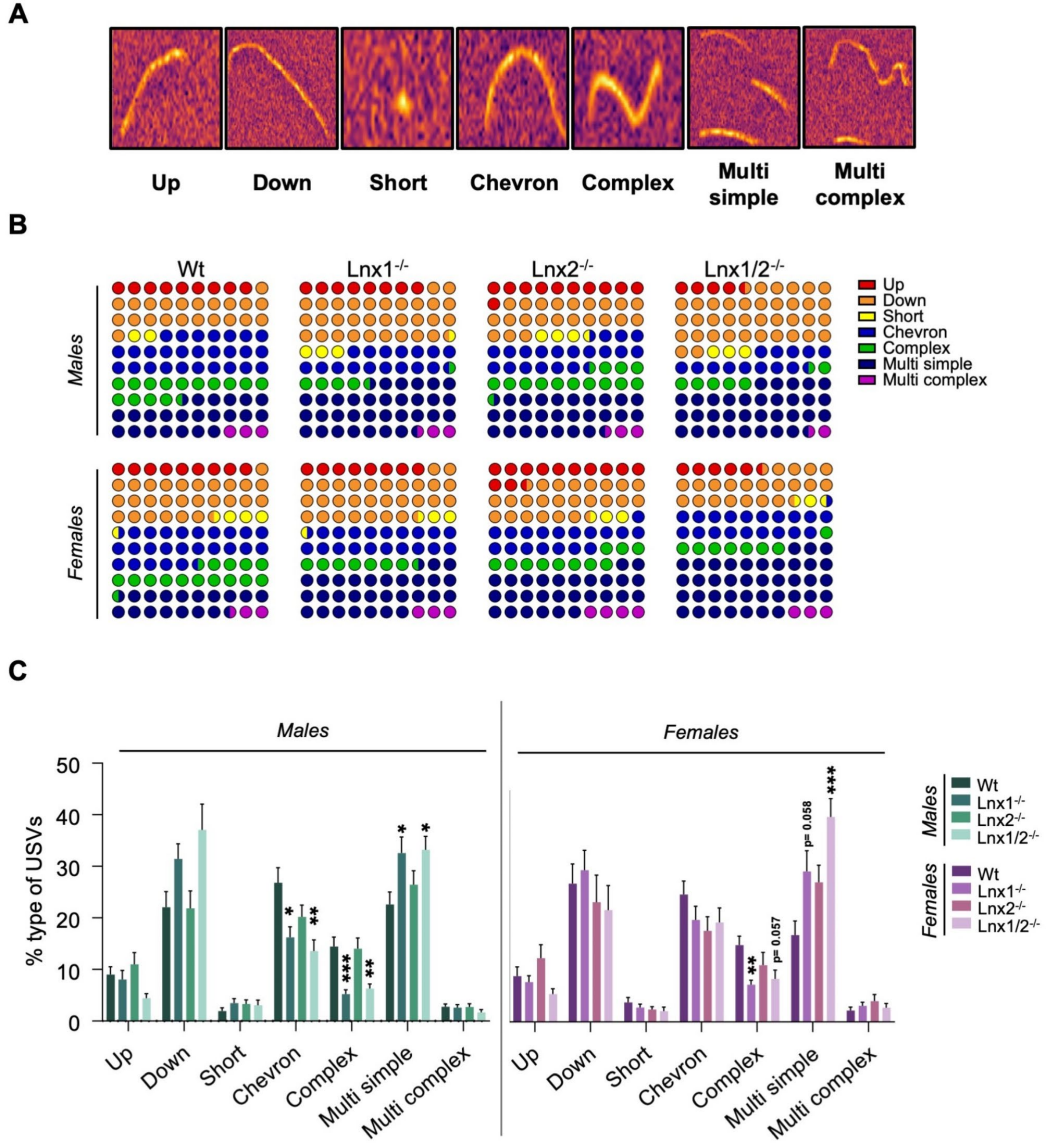
Vocal repertoire of of *Lnx* knockout mice **(A)** Representative spectrograms for seven categories of USV calls identified by supervised call classification using a neuronal network in the DeepSqueak software tool. **(B, C)** Dot plots and bar charts depicting the percentage of use of each category of USV call. **(C)** Data are presented as means +/- SEM; Statistical significance assessed by Kruskal-Wallis / Dunn’s multiple comparisons tests. *n* = 10–15 per group. *p < 0.05, **p < 0.01, ***p < 0.001

To gain greater insights into the vocal repertoires of *Lnx* knockout animals, USVs were classified into seven categories based on previous classification schemes [15, 25] with some modifications (Fig. 5A). The percentage of calls of each type for mice of each sex and genotype was then calculated (Fig. 5B, C). Male *Lnx1*^*−/−*^ and *Lnx1*^*−/−*^;*Lnx2*^*−/−*^ mice exhibited a significantly lower proportion of single component calls with either a chevron or complex structure and a concomitant higher proportion of multicomponent calls containing only simple call elements (*multi simple* calls). Similarly, for females, *Lnx1*^*−/−*^ and *Lnx1*^*−/−*^;*Lnx2*^*−/−*^ pups emitted a lower proportion of single component calls with complex structure and a higher proportion of *multi simple* calls compered to *Wt* mice. Overall, these results indicate that basic USV call properties in females and USV call repertoires in both sexes are altered in *Lnx1*^*−/−*^ and *Lnx1*^*−/−*^;*Lnx2*^*−/−*^ pups.

### Lnx1^−/−^ and Lnx1^−/−^;Lnx2^−/−^ mice exhibit lower body weight from postnatal through adult stages

**Fig. 6:**
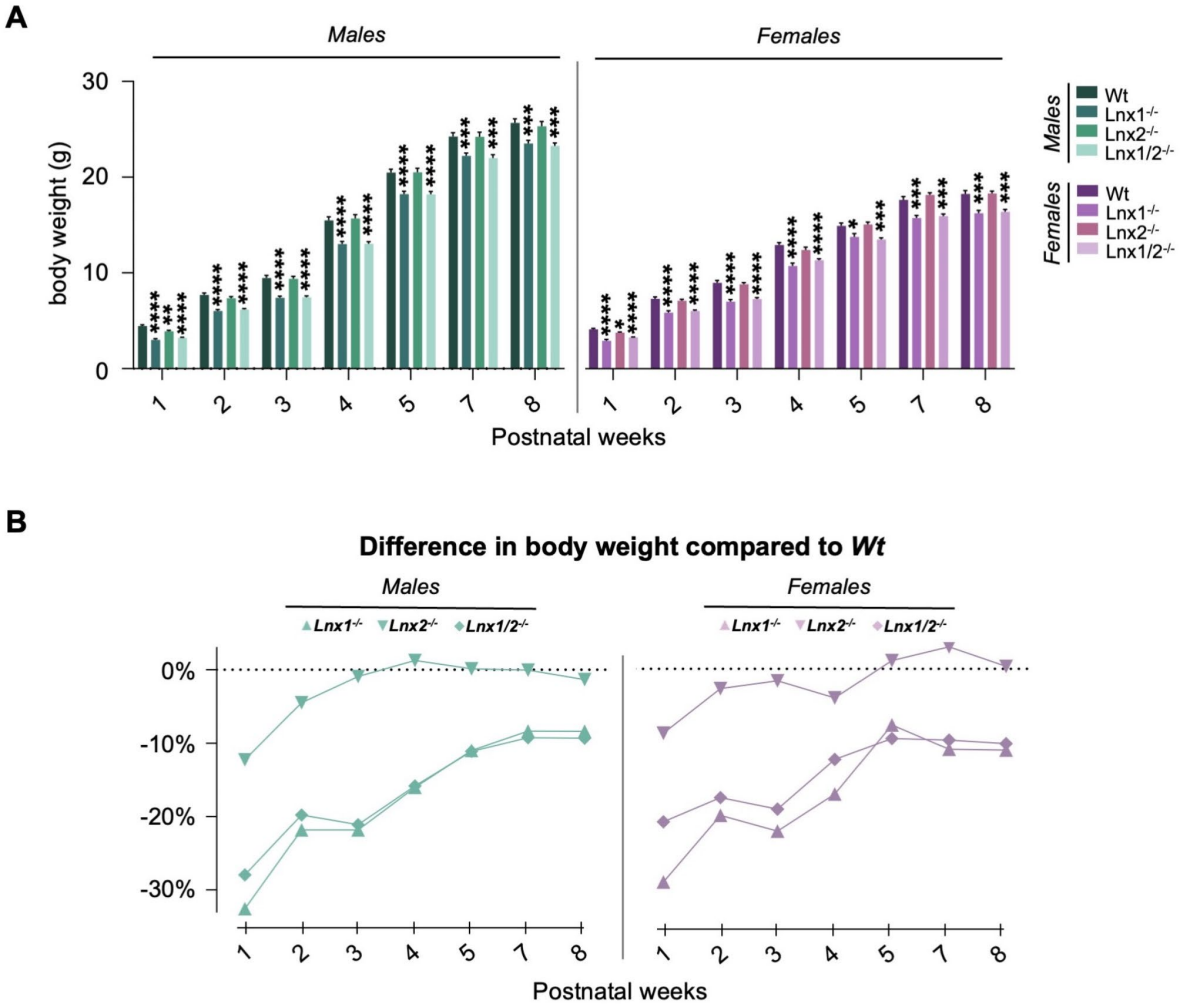
Influence of *Lnx* genotype on body weight **(A)** Body weights measured between one and eight weeks of age presented as means +/- SEM; differences between the indicated *Lnx* genotypes and wildtype animals were assessed using a two-way ANOVA with repeated measures performed on rank transformed data followed by a Dunnett’s multiple comparisons test. *p-values < 0.05, **p < 0.01, ***p < 0.001, ****p < 0.0001 *n* = 23–29 per group. **(B)** Mean body weight for mice of the indicated *Lnx* genotypes at each age expressed as a percentage difference in comparison to wildtype values

We previously noted that both male and female *Lnx1*^*−/−*^;*Lnx2*^*−/−*^ adult mice weighed approximately 10% less than their *Wt* counterparts [11]. To establish if this difference could be attributed to the loss of either LNX1 or LNX2 protein alone, *Lnx* knockout mice were weighed at weeks 1, 2, 3, 4, 5, 7 and 8. While both males and females of all three knockout genotypes weighed significantly less than *Wt* animals at one week of age, this was particularly pronounced for *Lnx1*^*−/−*^ and *Lnx1*^*−/−*^;*Lnx2*^*−/−*^ mice (Fig. 6A). This body weight difference for both sexes persisted through to week 8 for *Lnx1*^*−/−*^ and *Lnx1*^*−/−*^;*Lnx2*^*−/−*^ mice. At week one, the magnitude of these effects, in terms of percentage difference in body weight compared to *Wt* animals, were − 33% and − 29% for males and − 28% and − 21% for females for *Lnx1*^*−/−*^ and *Lnx1*^*−/−*^;*Lnx2*^*−/−*^ mice respectively (Fig. 6B). While by week eight these differences were − 8% and − 11% for males and − 9% and − 10% for females for *Lnx1*^*−/−*^ and *Lnx1*^*−/−*^;*Lnx2*^*−/−*^ mice respectively. By contrast, the initially lower body weights of one week old *Lnx2*^*−/−*^ mice (-12% for males and − 9% for females) were not significantly different from Wt animals from week two onwards.

## Discussion

LNX1 and LNX2 have the same overall domain structure and exhibit functional similarity in terms of interacting proteins and substrates for ubiquitination [6, 9, 27]. At the same time, LNX proteins are present at exceedingly low levels in most adult tissues, including the CNS. For these reasons, we previously examined the behaviour of *Lnx1*^*−/−*^;*Lnx2*^*−/−*^ mice in order to maximize the chance of observing a phenotype [11]. The most notable findings were decreased anxiety-related behaviour in the elevated plus maze for both males and females, and in the open field arena for male *Lnx1*^*−/−*^;*Lnx2*^*−/−*^ mice. One goal of the present study was to determine if these phenotypes could be attributed to the loss of either *Lnx* gene alone. We have recapitulated the findings of our previous study for *Lnx1*^*−/−*^;*Lnx2*^*−/−*^ mice in the elevated plus maze and open field tests. Notably, our mouse lines have been backcrossed onto a C57BL/6J genetic background for several more generations since the previous study and the colony moved to a new animal facility – supporting the robustness and veracity of this phenotype. However, no significant difference from *Wt* animals for either *Lnx1*^*−/−*^ or *Lnx2*^*−/−*^ single knockout mice was found in these two tests. In particular, in the elevated plus maze, neither single knockout cohort showed even a trend towards spending increased time in the open arms. These findings suggest that the loss of both *Lnx* genes is necessary to elicit decreased anxiety-related behaviour of *Lnx1*^*−/−*^;*Lnx2*^*−/−*^ double knockout mice in the open field and elevated plus maze, in agreement with the normal behaviour in these two paradigms that was reported for a distinct *Lnx1*^*−/−*^ single knockout line [12].

The possible influence of the observed reduced body weight of *Lnx1*^*−/−*^ and *Lnx1*^*−/−*^;*Lnx2*^*−/−*^ mice on locomotor activity in the elevated plus maze and open field tests merits consideration. However, at the time of behavioural testing the body weight difference is relatively small (approximately 10% less than wild-type animals) and previously we did not observe any motor deficits in double knockout mice in the rotorod and grip strength tests [11]. Additionally, total distance travelled in the open field, a measure of general locomotor activity, is comparable or increased for all Lnx knockout genotypes compared to wildtype animals. These observations indicate that general deficits in locomotor activity are not adversely influencing the analysis of *Lnx* knockout mice.

We also wanted to examine single and double knockout animals in other anxiety / risk-taking behavioural paradigms. Both the dark-light emergence and wire beam bridge tasks revealed significant changes in parameters indicative of either decreased anxiety or increased risk-taking for *Lnx2*^*−/−*^ and *Lnx1*^*−/−*^;*Lnx2*^*−/−*^, but not *Lnx1*^*−/−*^ mice. The prominent effect of *Lnx2* versus *Lnx1* knockout was especially noticeable in the wire beam bridge task for which *Lnx1*^*−/−*^ mice were very similar to *Wt* animals in terms of latency to both access and cross the bridge. The observations of increased risk-taking in these two tests represent the first behavioural phenotype reported for *Lnx2*^*−/−*^ single knockout mice.

The behavioural paradigms discussed above are all examples of exploratory-based approach–avoidance conflict tests that place the natural exploratory nature of mice in opposition to their innate tendencies to avoid open, brightly lit spaces or other risky scenarios. Conversely, marble burying task is a very different anxiety-related behavioural test in which the burying of marbles is interpreted as a proactive compulsive behaviour to relieve anxiety in response to an anxiogenic stimulus [16, 21]. No significant differences were seen for any *Lnx* knockout genotype in this task. Thus the phenotypes observed for adult *Lnx2*^*−/−*^ and *Lnx1*^*−/−*^;*Lnx2*^*−/−*^ seem to be quite specific to exploratory-based approach–avoidance conflict tests.

The wire beam bridge and dark-light emergence tasks might be regarding as assessing risk-taking more than anxiety, since the starting point for these tests is the safer environment of a covered enclosure. However, distinguishing reduced anxiety-like phenotypes versus increased impulsivity, novelty-seeking or risk-taking behaviour is difficult. Exploration of the two newly-introduced identical objects on day two of the novel object recognition test could be an indicator of novelty-seeking behaviour [24], however we did not observe any significant differences for *Lnx* knockout mice in this parameter. This argues against increased novelty seeking, however the exact nature of the decreased anxiety-related phenotype seen in *Lnx2*^*−/−*^ and *Lnx1*^*−/−*^;*Lnx2*^*−/−*^ requires further careful analysis.

The underlying basis for the distinct anxiety-related and risk-taking behavioural phenotypes of *Lnx1*^−/−^ and *Lnx2*^−/−^ mice at the molecular and neural circuitry level are not immediately obvious. Decreased anxiety-like behaviour in the elevated plus maze for double knockout but not for either single knockout line could be explained by functionally redundancy of LNX1 and LNX2 proteins, especially since they share some of the same interaction partners (reviewed in [6, 9]). However, several observations argue against this. Firstly, the expression patterns of their mRNAs in the nervous system are complementary both spatially and temporally [2, 28], calling into question the extent to which they are ever co-expressed in the same cells. Secondly, in contrast to LNX2, the neuronally expressed (p70 and p62) isoforms of LNX1 lack the catalytic RING domain [1, 10] and are presumed to be unable to directly mediate ubiquitination of substrate proteins. This suggests that LNX1 and LNX2 proteins may differentially regulate their interactions partners in neurons with LNX1 playing a stabilizing or anchoring role while LNX2 promotes ubiquitination and subsequent degradation. Thirdly, a number of LNX1-specific interacting proteins have been identified, including, for example, the presynaptic liprin-α proteins [7, 11]. These considerations, as well as the phenotypes observed for single knockout *Lnx1* and *Lnx2* animals in other tests, support the idea that LNX and LNX2 proteins play distinct, non-redundant roles, possibly in different brain regions and/or developmental stages.

Some insights into possible brain regions and developmental stages that mediate the influence of LNX proteins on behaviour can be gleaned by examining their expression patterns. *Lnx1* and *Lnx2* mRNAs are expressed in a largely complementary pattern during brain development with high *Lnx2* expression in the dorsal forebrain including the developing cortical plate, in comparison to more prominent expression of *Lnx1* in ventral forebrain structures [2]. By early postnatal stages *Lnx1* mRNA expression is seen across many fore-, mid- and hind brainregions, whereas *Lnx2* expression is restricted to the cerebral and cerebellar cortices. Thereafter *Lnx2* expression is dramatically downregulated, except in granule cell layers of both the olfactory bulb and the cerebellum. By contrast, *Lnx1* mRNA expression persists across many brain regions in adult mice [2, 28], most notably in the CA3 region of the hippocampus – albeit that LNX1 protein levels are very low [11]. In adult mice, *Lnx2* mRNA expression is minimal or absent in key brain regions that control anxiety such as the basolateral amygdala, the bed nucleus of the stria terminalis, the ventral hippocampus or the medial prefrontal cortex [2, 28, 29]. This suggests that the influence of LNX2 on anxiety and risk-taking behaviours is likely to be caused by subtle neural circuit defects established in the forebrain during development that then persist into adulthood. On the other hand, *Lnx1* mRNA expression in the postnatal and adult hippocampal CA3 region, implicate a possible function of LNX1 in the ventral hippocampus at the time of testing as contributing to decreased anxiety-related behaviours observed for double knockout animals in the elevated plus maze and open field. In terms of molecular mechanisms, the disruption of a LNX1-GluN2B-EphB2 ternary complex within CA3 that has been proposed to underlie defects in social memory in a different line of *Lnx1* null mice [12], might also play a role in changes in the anxiety-related phenotypes that we observed. The molecular pathways underlying LNX2’s effects on behavior are less clear, though interactions with presynaptic proteins (ERC/CAST [11, 30]), synaptic GTPase activating proteins (SYNGAP1, SRGAP2 [7, 11, 27]), gap junction proteins (Connexin36 [31]) and neurotransmitter transporters (GlyT2 [32]) are plausible candidates.

Liu et al. [12] performed extensive behavioural testing of a *Lnx1*^*−/−*^ single knockout line that is expected to lack all LNX1 protein isoforms (both neuronal and non-neuronal). They observed deficits in social memory, decreased sociality and increased social avoidance in these *Lnx1*^*−/−*^ mice at postnatal week 3. At this age they found that behaviour of *Lnx1*^*−/−*^ mice in the open field and elevated plus maze was indistinguishable from *Wt* animals as was learning and memory in the Y-maze, passive avoidance, fear conditioning and novel object recognition tasks. However, adult *Lnx1*^*−/−*^ mice (6 weeks old) were reported to exhibit impaired memory in the passive avoidance and fear conditioning tasks, as well as increased locomotor activity and repetitive jumping behaviour in the open field test [12]. Novel object recognition in adult animals was not assessed in their study. We show here that novel object recognition memory is intact at postnatal week 8 in our *Lnx1*^*−/−*^ mice as well as in *Lnx2*^*−/−*^ and *Lnx1*^*−/−*^;*Lnx2*^*−/−*^ animals. We also previously showed normal working memory in adult *Lnx1*^*−/−*^;*Lnx2*^*−/−*^ animals [11]. These observations suggest that adult *Lnx1*^*−/−*^ mice do not exhibit a general memory deficiency and that such deficits identified by Liu et al. may be restricted to social and fear memory. We also have not observed significantly increased locomotor activity or repetitive jumping behaviour for *Lnx1*^*−/−*^ mice in the open field test. This discrepancy may be related to the nature of the *Lnx1* gene knockout (knockout of neuronal specific isoforms only in our mice) or the genetic background of the mice (CD1 for Liu et al. [12] versus C57/BL6J for our mice).

Alterations in USVs have been increasingly used as a readout in mouse models of neurodevelopmental brain disorders, particularly those that are characterised by deficits in social communication [33, 34]. USVs of pups upon maternal separation are regarded as distress calls emitted in order to elicit retrieval to the nest. The number of USVs emitted by mouse pups in this paradigm follows a strain-specific developmental increase in the first postnatal week, decreasing thereafter as the pups become more independent [15, 25]. As an early form of social communication, number of USVs and other call properties in maternally-separated pups have been widely studied in mouse models of autism spectrum disorders. USV calling rate is often decreased, but sometimes increased, in these models – depending on the specific model and mutated gene (reviewed in [33, 35]). Altered USVs in pups can also correlate with anxiety-related behaviour in adults in certain contexts [36]. USVs in mouse pups thus serve as an early, albeit incompletely understood, readout of internal emotional or motivational states that can be informative about abnormalities in neurodevelopment [25, 26].

We did not observe *Lnx* genotype-dependent changes in the number of USVs for either sex. However, for females, but not males, significant differences in call length, delta frequency, principal frequency and power of vocalizations were apparent for *Lnx1*^*−/−*^;*Lnx2*^*−/−*^ mice in comparison to *Wt* controls. The power of vocalizations was also higher for female *Lnx1*^*−/−*^ single knockouts. Classification of calls to examine the vocal repertoire of *Lnx* knockout mice revealed prominent differences for *Lnx1*^*−/−*^ and *Lnx1*^*−/−*^;*Lnx2*^*−/−*^ mice of both sexes in their use of several categories of call type. While the relevance or meaning of these alterations in vocal repertoire in terms of pup-dam communication are not clear, they are indicative of neurodevelopmental abnormalities that are apparent as early as postnatal day 9. Notably, *Lnx1*^*−/−*^ and *Lnx1*^*−/−*^;*Lnx2*^*−/−*^ (and to a lesser extent *Lnx2*^*−/−*^) pups weighed significantly less than *Wt* counterparts at this age. Thus it is possible that differences in USVs are reflective of differences in body weight and a general developmental delay in *Lnx1*^*−/−*^ and *Lnx1*^*−/−*^;*Lnx2*^*−/−*^ mice that could, for example, cause altered laryngeal morphology or ability to control breathing with consequent changes in USVs [33]. However, the observation of altered vocal repertoires in male *Lnx1*^*−/−*^ and *Lnx1*^*−/−*^;*Lnx2*^*−/−*^ mice, in the absence of significant differences in basic call parameters (that are known to change developmentally in a characteristic manner [15, 25, 37]), would argue against these changes being purely a consequence of a general developmental delay and the physically smaller size of these mice. Since the *Lnx1*^*−/−*^ mice in this study lack only the neuronal isoforms of *Lnx1*, both the body weight differences and alterations in USVs observed in *Lnx1*^*−/−*^ must be a consequence, either directly or indirectly, of a neurodevelopmental defect.

## Conclusions

In summary, the analysis presented here delineates for the first time distinct roles for LNX1 and LNX2 proteins in regulating anxiety-related and risk-taking behaviour, ultrasonic vocalizations and body weight. This provides the basis for future mechanistic studies of these phenomena.

## Electronic supplementary material

Below is the link to the electronic supplementary material.

Supplementary Material 1

Supplementary Material 2

## Data Availability

The datasets used and/or analysed during the current study are available from the corresponding author on reasonable request.

## Abbreviations

LNX: Ligand of numb protein X
CNS: Central Nervous System
PDZ: PSD-95, DlgA, ZO-1
RING: Really Interesting New Gene
SIH: Stress-induced Hyperthermia
USV: Ultrasonic Vocalisation

## References

[1] Dho SE, Jacob S, Wolting CD, French MB, Rohrschneider LR, McGlade CJ. The mammalian numb phosphotyrosine-binding domain. Characterization of binding specificity and identification of a novel PDZ domain-containing numb binding protein, LNX. J Biol Chem. 1998;273(15):9179–87. 9535908 10.1074/jbc.273.15.9179

[2] Rice DS, Northcutt GM, Kurschner C. The Lnx family proteins function as molecular scaffolds for numb family proteins. Mol Cell Neurosci. 2001;18(5):525–40. 11922143 10.1006/mcne.2001.1024

[3] Nie J, Li SS, McGlade CJ. A novel PTB-PDZ domain interaction mediates isoform-specific ubiquitylation of mammalian numb. J Biol Chem. 2004;279(20):20807–15. 14990566 10.1074/jbc.M311396200

[4] Nie J, McGill MA, Dermer M, Dho SE, Wolting CD, McGlade CJ. LNX functions as a RING type E3 ubiquitin ligase that targets the cell fate determinant numb for ubiquitin-dependent degradation. EMBO J. 2002;21(12):93–102. 11782429 10.1093/emboj/21.1.93 PMC125803

[5] Nayak D, Sivaraman J. Structural basis for the indispensable role of a unique zinc finger motif in LNX2 ubiquitination. Oncotarget. 2015;6(33):34342–57. 26451611 10.18632/oncotarget.5326 PMC4741457

[6] Hong J, Won M, Ro H. The molecular and pathophysiological functions of members of the LNX/PDZRN E3 ubiquitin ligase family. Molecules 2020, 25(24). 10.3390/molecules25245938 PMC7765395 33333989

[7] Lenihan JA, Saha O, Young PW. Proteomic analysis reveals novel ligands and substrates for LNX1 E3 ubiquitin ligase. PLoS ONE. 2017;12(11):e0187352. 29121065 10.1371/journal.pone.0187352 PMC5679597

[8] Wolting CD, Griffiths EK, Sarao R, Prevost BC, Wybenga-Groot LE, McGlade CJ. Biochemical and computational analysis of LNX1 interacting proteins. PLoS ONE. 2011;6(11):e26248. 22087225 10.1371/journal.pone.0026248 PMC3210812

[9] Young PW. LNX1/LNX2 proteins: functions in neuronal signaling and beyond. Neuronal Signal. 2018;2(2):1–20. 10.1042/NS20170191 PMC7373230 32714586

[10] Lenihan JA, Saha O, Mansfield LM, Young PW. Tight, cell type-specific control of LNX expression in the nervous system, at the level of transcription, translation and protein stability. Gene. 2014;552(1):39–50. 25200495 10.1016/j.gene.2014.09.011

[11] Lenihan JA, Saha O, Heimer-McGinn V, Cryan JF, Feng G, Young PW. Decreased Anxiety-Related behaviour but apparently unperturbed NUMB function in ligand of NUMB Protein-X (LNX) 1/2 double knockout mice. Mol Neurobiol. 2017;54(10):8090–109. 27889896 10.1007/s12035-016-0261-0

[12] Liu XD, Ai PH, Zhu XN, Pan YB, Halford MM, Henkemeyer M, Feng DF, Xu TL, Sun S, Xu NJ. Hippocampal Lnx1-NMDAR multiprotein complex mediates initial social memory. Mol Psychiatry 2019. 10.1038/s41380-019-0606-y PMC8550978 31772302

[13] Liu XD, Zhu XN, Halford MM, Xu TL, Henkemeyer M, Xu NJ. Retrograde regulation of mossy fiber axon targeting and terminal maturation via postsynaptic Lnx1. J Cell Biol. 2018;217(11):4007–24. 30185604 10.1083/jcb.201803105 PMC6219728

[14] Sigmon JS, Blanchard MW, Baric RS, Bell TA, Brennan J, Brockmann GA, Burks AW, Calabrese JM, Caron KM, Cheney RE, et al. Content and performance of the MiniMUGA genotyping array: A new tool to improve rigor and reproducibility in mouse research. Genetics. 2020;216(4):905–30. 33067325 10.1534/genetics.120.303596 PMC7768238

[15] Scattoni ML, Gandhy SU, Ricceri L, Crawley JN. Unusual repertoire of vocalizations in the BTBR T + tf/J mouse model of autism. PLoS ONE. 2008;3(8):e3067. 18728777 10.1371/journal.pone.0003067 PMC2516927

[16] Cryan JF, Holmes A. The ascent of mouse: advances in modelling human depression and anxiety. Nat Rev Drug Discov. 2005;4(9):775–90. 16138108 10.1038/nrd1825

[17] Liu GX, Cai GQ, Cai YQ, Sheng ZJ, Jiang J, Mei Z, Wang ZG, Guo L, Fei J. Reduced anxiety and depression-like behaviors in mice lacking GABA transporter subtype 1. Neuropsychopharmacology. 2007;32(7):1531–9. 17164814 10.1038/sj.npp.1301281

[18] Smith GW, Aubry JM, Dellu F, Contarino A, Bilezikjian LM, Gold LH, Chen R, Marchuk Y, Hauser C, Bentley CA, et al. Corticotropin releasing factor receptor 1-deficient mice display decreased anxiety, impaired stress response, and aberrant neuroendocrine development. Neuron. 1998;20(6):1093–102. 9655498 10.1016/s0896-6273(00)80491-2

[19] Bortolato M, Godar SC, Davarian S, Chen K, Shih JC. Behavioral disinhibition and reduced anxiety-like behaviors in monoamine oxidase B-deficient mice. Neuropsychopharmacology. 2009;34(13):2746–57. 19710633 10.1038/npp.2009.118 PMC2783894

[20] Mosher LJ, Godar SC, Morissette M, McFarlin KM, Scheggi S, Gambarana C, Fowler SC, Di Paolo T, Bortolato M. Steroid 5alpha-reductase 2 deficiency leads to reduced dominance-related and impulse-control behaviors. Psychoneuroendocrinology. 2018;91:95–104. 29544191 10.1016/j.psyneuen.2018.02.007 PMC5901899

[21] Njung’e K, Handley SL. Evaluation of marble-burying behavior as a model of anxiety. Pharmacol Biochem Behav. 1991;38(1):63–7. 2017455 10.1016/0091-3057(91)90590-x

[22] Olivier B, Zethof T, Pattij T, van Boogaert M, van Oorschot R, Leahy C, Oosting R, Bouwknecht A, Veening J, van der Gugten J, et al. Stress-induced hyperthermia and anxiety: Pharmacological validation. Eur J Pharmacol. 2003;463(1–3):117–32. 12600705 10.1016/s0014-2999(03)01326-8

[23] Gulinello M, Mitchell HA, Chang Q, Timothy O’Brien W, Zhou Z, Abel T, Wang L, Corbin JG, Veeraragavan S, Samaco RC, et al. Rigor and reproducibility in rodent behavioral research. Neurobiol Learn Mem. 2019;165:106780. 29307548 10.1016/j.nlm.2018.01.001 PMC6034984

[24] Dulawa SC, Grandy DK, Low MJ, Paulus MP, Geyer MA. Dopamine D4 receptor-knock-out mice exhibit reduced exploration of novel stimuli. J Neurosci. 1999;19(21):9550–6. 10531457 10.1523/JNEUROSCI.19-21-09550.1999 PMC6782928

[25] Heckman J, McGuinness B, Celikel T, Englitz B. Determinants of the mouse ultrasonic vocal structure and repertoire. Neurosci Biobehav Rev. 2016;65:313–25. 27060755 10.1016/j.neubiorev.2016.03.029

[26] Karigo T. Gaining insights into the internal States of the rodent brain through vocal communications. Neurosci Res. 2022;184:1–8. 35908736 10.1016/j.neures.2022.07.008

[27] Flynn M, Saha O, Young P. Molecular evolution of the LNX gene family. BMC Evol Biol. 2011;11:235. 21827680 10.1186/1471-2148-11-235 PMC3162930

[28] Allen Brain Atlas (https://mouse.brain-map.org).

[29] Calhoon GG, Tye KM. Resolving the neural circuits of anxiety. Nat Neurosci. 2015;18(10):1394–404. 26404714 10.1038/nn.4101 PMC7575249

[30] Higa S, Tokoro T, Inoue E, Kitajima I, Ohtsuka T. The active zone protein CAST directly associates with Ligand-of-Numb protein X. Biochem Biophys Res Commun. 2007;354(3):686–92. 17257582 10.1016/j.bbrc.2007.01.036

[31] Lynn BD, Li X, Hormuzdi SG, Griffiths EK, McGlade CJ, Nagy JI. E3 ubiquitin ligases LNX1 and LNX2 localize at neuronal gap junctions formed by connexin36 in rodent brain and molecularly interact with connexin36. Eur J Neurosci. 2018;48(9):3062–81. 30295974 10.1111/ejn.14198

[32] de la Rocha-Munoz A, Nunez E, Arribas-Gonzalez E, Lopez-Corcuera B, Aragon C, de Juan-Sanz J. E3 ubiquitin ligases LNX1 and LNX2 are major regulators of the presynaptic glycine transporter GlyT2. Sci Rep. 2019;9(1):14944. 31628376 10.1038/s41598-019-51301-x PMC6802383

[33] Premoli M, Pietropaolo S, Wohr M, Simola N, Bonini SA. Mouse and rat ultrasonic vocalizations in neuroscience and neuropharmacology: state of the Art and future applications. Eur J Neurosci. 2023;57(12):2062–96. 36889803 10.1111/ejn.15957

[34] Scattoni ML, Crawley J, Ricceri L. Ultrasonic vocalizations: a tool for behavioural phenotyping of mouse models of neurodevelopmental disorders. Neurosci Biobehav Rev. 2009;33(4):508–15. 18771687 10.1016/j.neubiorev.2008.08.003 PMC2688771

[35] Caruso A, Ricceri L, Scattoni ML. Ultrasonic vocalizations as a fundamental tool for early and adult behavioral phenotyping of autism spectrum disorder rodent models. Neurosci Biobehav Rev. 2020;116:31–43. 32544538 10.1016/j.neubiorev.2020.06.011

[36] Budylin T, Guariglia SR, Duran LI, Behring BM, Shaikh Z, Neuwirth LS, Banerjee P. Ultrasonic vocalization sex differences in 5-HT(1A)-R deficient mouse pups: predictive phenotypes associated with later-life anxiety-like behaviors. Behav Brain Res. 2019;373:112062. 31288061 10.1016/j.bbr.2019.112062

[37] Wiaderkiewicz J, Glowacka M, Grabowska M, Jaroslaw-Jerzy B. Ultrasonic vocalizations (USV) in the three standard laboratory mouse strains: developmental analysis. Acta Neurobiol Exp (Wars). 2013;73(4):557–63. 24457645 10.55782/ane-2013-1959

