# Supplementary material for: Differential neuronal functions of LNX1 and LNX2 revealed by behavioural analysis in single and double knockout mice
Source: Behav Brain Funct. 2025 Apr 23;21:13. doi: 10.1186/s12993-025-00276-z (PMC12020136; doi:10.1186/s12993-025-00276-z)
Supplement: Supplementary file 1 — Supplementary Material 1 [file 12993_2025_276_MOESM1_ESM.pdf]

Additional File 1: Statistical analysis to test for effects of sex in each behavioural paradigm

Tests with P vaues that reach statistical significance are highlighted in bold and underlined

			**Wt**	**$Lnx1^{-/-}$**	**$Lnx2^{-/-}$**	**$Lnx1/2^{-/-}$**
**ELEVATED PLUS MAZE**	n	Male	15	15	15	15
		Female	14	15	14	14
	% Open Arm Entry	*Mann-Whitney P value*	0.7552	0.1758	0.3703	0.755
		Mann-Whitney U	97.5	79.5	84	97.5
		Median male	23.53	$^{\#}$31.82	35.71	38.89
		Median female	28.95	21.43	28.59	41.67
	Time Open Arms	*Mann-Whitney P value*	0.5325	0.2169	0.8717	0.8132
		Mann-Whitney U	90	82	101	99
		Median male	48.10	51.90	45.70	78.60
		Median female	47.00	$^{\#}$25.50	48.95	$^{\#\#\#}$80.15
	Time Close Arms	*Mann-Whitney P value*	0.5325	0.3453	0.3536	0.8805
		Mann-Whitney U	90	89	83	101
		Median male	242.00	236.30	224.80	211.30
		Median female	242.90	267.00	240.10	$^{\#\#}$211.10

			**Wt**	**$Lnx1^{-/-}$**	**$Lnx2^{-/-}$**	**$Lnx1/2^{-/-}$**
**OPEN FIELD**	n	Male	15	15	15	15
		Female	14	15	14	14
	0-5 minutes/5-10 minutes					
	Time in the centre	*Mann-Whitney P value*	0.7892/0.8132	0.1064/0.2169	0.9829/0.525	0.0954/0.1718
		Mann-Whitney U	98.5/99	73/82	104/90	66.5/73
		Median male	$^{\#}$56.30/67.20	60.10/69.60	57.50/77.50	56.90/99.50
		Median female	56.25/$^{\#}$49.25	75.90/101.70	57.50/92.30	47.95/76.65
	Time in the corners	*Mann-Whitney P value*	0.5045/0.9572	0.2496/0.1485	0.9486/0.683	**0.0091**/0.0511
		Mann-Whitney U	89/103.5	84/77	103/95	46/60
		Median male	97.60/$^{\#\#}$87.50	99.40/91.00	95.00/78.50	95.60/75.00
		Median female	86.00/87.30	85.00/$^{\#}$77.40	99.15/79.70	109.20/93.80
	Entries in the centre	*Mann-Whitney P value*	0.6577/0.3916	0.1168/0.7815	0.443/0.2185	**0.0014**/0.5378
		Mann-Whitney U	94.5/85	74.5/105.5	87/76.5	34.5/90.5
		Median male	15.00/$^{\#\#}$11.00	15.00/12.00	15.00/14.00	18.00/12.00
		Median female	14.50/10.50	18.00/11.00	$^{\#}$16.50/$^{\#}$10.50	11.50/12.00
	Entries in the corners	*Unpaired t test_P value*	0.147/0.7598	0.4823/0.8648	0.6277/0.3620	0.136/0.5044
		Mean male	21.33/19.27	24.20/19.53	25.13/19.80	25.67/19.13
		Mean female	26.50/18.57	22.60/19.20	26.43/18.50	30.07/20.64
	Distance	*Unpaired t test_P value*	0.1284/0.792	0.9001/0.5243	0.2546/0.3478	0.7622/0.9141
		Mean male	13.91/11.88	15.21/12.58	16.17/13.11	17.77/12.87
		Mean female	16.74/11.52	15.36/11.88	18.13/12.21	18.37/12.74

Test	Measure		Wt	$Lnx1^{-/-}$	$Lnx2^{-/-}$	$Lnx1/2^{-/-}$
DARK TO LIGHT EMERGENCE TEST	n	Male	15	15	14	15
		Female	15	14	13	15
	Latency to emerge	*Mann-Whitney P value*	0.9435	0.5833	0.2642	0.8381
		Mann-Whitney U	110.5	92	67.5	107
		Median male	[####]16.70	[####]12.80	[###]19.00	[####]14.20
		Median female	[####]18.60	[##]17.50	[####]14.80	[###]14.50
	Time in open area	*Mann-Whitney P value*	0.6164	0.4773	0.402	0.8063
		Mann-Whitney U	100	88	73	106
		Median male	130.60	180.20	[#]199.50	188.20
		Median female	128.80	146.00	[##]234.00	189.70
	Number of transitions	*Unpaired t test_P value*	0.217	0.0909	0.6635	0.3041
		Mean male	12.60	14.93	14.57	17.53
		Mean female	16.13	17.71	15.54	20.13

Test	Measure		Wt	$Lnx1^{-/-}$	$Lnx2^{-/-}$	$Lnx1/2^{-/-}$
WIRE BEAM BRIDGE	n	Male	15	15	13	15
		Female	15	14	13	15
	Latency to access the bridge	*Mann-Whitney P value*	0.8058	0.3088	0.5534	0.6132
		Mann-Whitney U	106	81	72.5	93
		Median male	[#]250.00	193	[##]164.00	[#]127.00
		Median female	282.00	303.00	113.00	103.50
	Latency to cross the bridge	*Mann-Whitney P value*	0.8935	0.4551	0.2175	0.2169
		Mann-Whitney U	109	87.5	60	76
		Median male	[#]265.00	[#]209.00	222	[#]179.00
		Median female	291.00	342.00	148.00	[##]114.50

Test	Measure		Wt	$Lnx1^{-/-}$	$Lnx2^{-/-}$	$Lnx1/2^{-/-}$
MARBLE BURYING	n	Male	15	15	15	15
		Female	14	15	14	14
	Number of marbles buried	*Mann-Whitney P value*	**0.023**	0.9755	0.0783	**0.0016**
		Mann-Whitney U	53.5	111.5	64.5	35.5
		Median male	[##]17	11	14	[##]-10.5
		Median female	12.25	12.50	8.75	-7.50

Test	Measure		Wt	$Lnx1^{-/-}$	$Lnx2^{-/-}$	$Lnx1/2^{-/-}$
STRESS INDUCED HYPERTHERMIA	n	Male	15	15	15	15
		Female	15	14	12	15
	Change in temperature	*Mann-Whitney P value*	0.143	**0.0063**	**0.0273**	**0.0204**
		Mann-Whitney U	77	44	41.5	62
		Median male	1.5	[#]1.6	1.4	1.5
		Median female	[#]1.3	1.20	0.70	1.05

			Wt	Lnx1$^{-/-}$	Lnx2$^{-/-}$	Lnx1/2$^{-/-}$
NOVEL OBJECT	Day2					
	n	Male	15	15	14	15
		Female	15	14	13	16
	Interaction time day 2	*Unpaired t test_P value*	0.7467	0.3	0.8231	0.7167
		Mean male	85.93	88.33	96.89	99.35
		Mean female	90.71	99.29	99.81	104.2
	Day3					
	n	Male	12	13	13	15
		Female	14	13	12	15
	familiar/novel					
	Interaction time day 3	*Unpaired t test_P value*	0.5981/0.8791	0.0529/0.4256	0.8131/0.1286	0.2329/0.5032
		Mean male	34.286/48.901	33.308/55.162	40.261/58.627	43.943/59.385
		Mean female	36.272/50.239	48.417/61.717	41.756/72.757	54.420/65.135
	Discrimi-nation index	*Unpaired t test_P value*	0.8396	0.2042	0.7155	0.9322
		Mean male	56.85	63.09	59.22	56.36
		Mean female	57.9	56.14	60.84	55.96

			Wt	Lnx1$^{-/-}$	Lnx2$^{-/-}$	Lnx1/2$^{-/-}$
ULTRASONIC VOCALISATION	n	Male	16	17	15	12
		Female	16	16	13	12
	Number of USVs	*Mann-Whitney P value*	0.6621	0.8381	0.8295	0.64
		Mann-Whitney U	116	130	92.5	63.5
		Median male	#109.5	121.00	124.00	135.00
		Median female	#91.00	121.00	130.00	141.00
	n	Male	16	17	14	12
		Female	16	15	13	11
	USVs length	*Unpaired t test_P value*	**0.0101**	0.6601	0.8682	0.0658
		Mean male	0.03568	0.03378	0.03076	0.03140
		Mean female	0.30020	0.32420	0.03126	0.03786
	Principal frequency	*Unpaired t test_P value*	**0.0100**	0.0859	0.7999	0.7489
		Mean male	81.48	78.44	83.19	78.87
		Mean female	86.44	82.03	82.61	79.29
	Delta frequency	*Unpaired t test_P value*	**0.0197**	0.6697	0.4828	0.0741
		Mean male	21.34000	21.05000	20.02000	20.73000
		Mean female	17.08000	20.19000	21.60000	25.45000
	Mean power	*Unpaired t test_P value*	**0.0201**	0.5134	0.3951	**0.0235**
		Mean male	-85.36	-84.20	-86.49	-84.82
		Mean female	-87.31	-84.79	-85.58	-82.97
	Latency	*Mann-Whitney P value*	0.626	0.667	0.4559	0.6277
		Mann-Whitney U	107	101	74	52
		Median male	####3.068	####2.349	####-1.638	##2.364
		Median female	###4.354	##4.194	####-1.264	##3.419

# indicates data that is not normally distributed according to the Shapiro-Wilk test. #P ≤ 0.05, ##P ≤ 0.01 and ###P ≤ 0.001
