# Supplementary material for: Differential neuronal functions of LNX1 and LNX2 revealed by behavioural analysis in single and double knockout mice
Source: Behav Brain Funct. 2025 Apr 23;21:13. doi: 10.1186/s12993-025-00276-z (PMC12020136; doi:10.1186/s12993-025-00276-z)
Supplement: Supplementary file 2 — Supplementary Material 2 [file 12993_2025_276_MOESM2_ESM.pdf]

Additional File 2: Statistical analysis to test for effects of Lnx genotype in each behavioural paradigm

Tests with P vaues that reach statistical significance are highlighted in bold and underlined (values that are very close to 0.05 are underlined only)

Paradigm	Measure	Test	Sex	P value	K-W statistic	Post-hoc test	Comparison	Adjusted P value	Mean rank 1	Mean rank 2	Mean rank diff.	n1	n2
**ELEVATED PLUS MAZE**	% Open Arm Entry	Kruskal-Wallis test	Sexes Combined	**<0.0001**	22.16	Dunn's mult. comp. test	*Wt* vs. *Lnx1*$^{-/-}$	>0.9999	46.03	50.7	-4.67	29	30
							Wt vs. *Lnx2*$^{-/-}$	0.8606	46.03	55.52	-9.48	29	29
							Wt vs. *Lnx1/2*$^{-/-}$	**<0.0001**	46.03	84.03	-38.00	29	29
	Time Open Arms	Kruskal-Wallis test	Sexes Combined	**0.0034**	13.64	Dunn's mult. comp. test	*Wt* vs. *Lnx1*$^{-/-}$	>0.9999	53.71	49.38	4.32	29	30
							Wt vs. *Lnx2*$^{-/-}$	>0.9999	53.71	54.34	-0.64	29	29
							Wt vs. *Lnx1/2*$^{-/-}$	**0.0141**	53.71	78.9	-25.19	29	29
	Time Close Arms	Kruskal-Wallis test	Sexes Combined	**0.017**	10.19	Dunn's mult. comp. test	*Wt* vs. *Lnx1*$^{-/-}$	>0.9999	66.53	64.42	2.12	29	30
							Wt vs. *Lnx2*$^{-/-}$	>0.9999	66.53	63.17	3.36	29	29
							Wt vs. *Lnx1/2*$^{-/-}$	**0.0159**	66.53	41.69	24.84	29	29

0-5 minutes/5-10 minutes

Paradigm	Measure	Test	Sex	P value	K-W statistic	Post-hoc test	Comparison	Adjusted P value	Mean rank 1	Mean rank 2	Mean rank diff.	n1	n2
**OPEN FIELD**	Time in the centre	Kruskal-Wallis test	Males	0.9675/0.0576	0.2594/7.497	Dunn's mult. comp. test	*Wt* vs. *Lnx1*$^{-/-}$	>0.9999/>0.9999	29.4/23.13	32.17/27.00	2.77/3.87	15	15
							Wt vs. *Lnx2*$^{-/-}$	>0.9999/0.4562	29.4/23.13	29.47/32.27	0.07/9.13	15	15
							Wt vs. *Lnx1/2*$^{-/-}$	>0.9999/**0.0295**	29.4/23.13	30.97/39.60	1.57/16.47	15	15
			Females	0.1911/0.1753	4.75/4.953		*Wt* vs. *Lnx1*$^{-/-}$	0.4988/0.1349	27.93/21.50	36.47/33.87	8.54/12.37	14	15
							Wt vs. *Lnx2*$^{-/-}$	>0.9999/0.2215	27.93/21.50	27.61/32.71	-0.32/11.21	14	14
							Wt vs. *Lnx1/2*$^{-/-}$	>0.9999/0.9994	27.93/21.50	23.46/27.57	-4.46/6.07	14	14
	Time in the corners	Kruskal-Wallis test	Males	0.9850/0.1482	0.1518/5.344	Dunn's mult. comp. test	*Wt* vs. *Lnx1*$^{-/-}$	>0.9999/>0.9999	31.07/33.93	31.57/35.20	0.5/1.27	15	15
							Wt vs. *Lnx2*$^{-/-}$	>0.9999/>0.9999	31.07/33.93	30.07/31.00	-1/-2.93	15	15
							Wt vs. *Lnx1/2*$^{-/-}$	>0.9999/0.1754	31.07/33.93	29.3/21.87	-1.77/-12.07	15	15
			Females	0.0744/05147	6.923/2.289		*Wt* vs. *Lnx1*$^{-/-}$	>0.9999/0.9556	26.71/31.29	23.13/25.13	-3.58/-6.15	14	15
							Wt vs. *Lnx2*$^{-/-}$	>0.9999/>0.9999	26.71/31.29	27.93/26.61	1.21/-4.68	14	14
							Wt vs. *Lnx1/2*$^{-/-}$	0.1717/>0.9999	26.71/31.29	38.64/33.25	11.93/1.96	14	14

**OPEN FIELD**	Entries in the centre	Kruskal-Wallis test		*P value*	*K-W statistic*	Dunn's mult. comp. test		*Adjusted P value*	*Mean rank 1*	*Mean rank 2*	*Mean rank diff.*	*n1*	*n2*
			Males	0.0957/0.5470	6.351/2.124		*Wt* vs. *Lnx1*$^{-/-}$	>0.9999/>0.9999	24.27/26.83	28.5/27.73	4.23/0.9	15	15
							Wt vs. *Lnx2*$^{-/-}$	>0.9999/0.6939	24.27/26.83	29.53/34.43	5.27/7.6	15	15
							Wt vs. *Lnx1/2*$^{-/-}$	<u>**0.0457**</u>/0.9943	24.27/26.83	39.7/33.00	15.43/6.17	15	15
			Females	<u>**0.0335**</u>/0.7078	8.703/1.390		*Wt* vs. *Lnx1*$^{-/-}$	0.675/>0.9999	27.07/25.43	34.53/28.73	-7.46/-3.30	14	15
							Wt vs. *Lnx2*$^{-/-}$	0.6272/>0/9999	27.07/25.43	34.93/29.07	-7.86/-3.64	14	14
							Wt vs. *Lnx1/2*$^{-/-}$	0.6027/0.7177	27.07/25.43	19.07/32.79	8/-7.36	14	14
	Entries in the corners	ANOVA summary		*P value*	*F-statistic*	Dunnett's mult. comp. test		*Adjusted P value*	*Mean 1*	*Mean 2*	*Mean diff.*	*n1*	*n2*
			Males	0.321/0.9807	1.192/0.0597		*Wt* vs. *Lnx1*$^{-/-}$	0.5271/0.9973	21.33/19.27	24.2/19.53	2.87/0.27	15	15
							Wt vs. *Lnx2*$^{-/-}$	0.305/0.9796	21.33/19.27	25.13/19.80	3.8/0.53	15	15
							Wt vs. *Lnx1/2*$^{-/-}$	0.2104/0.9997	21.33/19.27	25.67/19.13	4.33/-0.13	15	15
			Females	0.1388/0.3841	1.912/0.3841		*Wt* vs. *Lnx1*$^{-/-}$	0.4591/0.9845	26.5/18.57	22.6/19.20	-3.9/0.63	14	15
							Wt vs. *Lnx2*$^{-/-}$	>0.9999/>0.9999	26.5/18.57	26.43/18.50	-0.07/-0.07	14	14
							Wt vs. *Lnx1/2*$^{-/-}$	0.5408/0.6861	26.5/18.57	30.07/20.64	3.57/2.07	14	14
	Distance	ANOVA summary		*P value*	*F-statistic*	Dunnett's mult. comp. test		*Adjusted P value*	*Mean 1*	*Mean 2*	*Mean diff.*	*n1*	*n2*
			Males	0.0817/0.4546	2.355/0.4546		*Wt* vs. *Lnx1*$^{-/-}$	0.7205/0.8666	13.91/11.88	15.21/12.58	1.3/0.7	15	15
							Wt vs. *Lnx2*$^{-/-}$	0.3129/0.5570	13.91/11.88	16.17/13.11	2.26/1.24	15	15
							Wt vs. *Lnx1/2*$^{-/-}$	<u>**0.0338**</u>/0.7079	13.91/11.88	17.77/12.87	3.86/0.99	15	15
			Females	0.3369/0.3728	1.152/0.3728		*Wt* vs. *Lnx1*$^{-/-}$	0.7931/0.9797	16.74/11.52	15.36/11.88	-1.38/0.37	14	15
							Wt vs. *Lnx2*$^{-/-}$	0.7977/0.889	16.74/11.52	18.13/12.21	1.39/0.7	14	14
							Wt vs. *Lnx1/2*$^{-/-}$	0.7157/0.6158	16.74/11.52	18.37/12.74	1.63/1.22	14	14

DARK TO LIGHT EMERGENCE TEST

Measure	Test	Sex	P value	Statistic	Post hoc test	Comparison	Adjusted P value	Mean rank 1	Mean rank 2	Mean rank diff.	n1	n2
Latency to emerge	Kruskal-Wallis test	Sexes Combined	*P value*	*K-W statistic*	Dunn's mult. comp. test		*Adjusted P value*	*Mean rank 1*	*Mean rank 2*	*Mean rank diff.*	*n1*	*n2*
			0.338	3.37		*Wt* vs. *Lnx1*$^{-/-}$	0.6466	64.47	53.62	10.85	30	29
						Wt vs. *Lnx2*$^{-/-}$	>0.9999	64.47	64.13	0.337	30	27
						Wt vs. *Lnx1/2*$^{-/-}$	0.4715	64.47	52.18	12.28	30	30
Time in open area	Kruskal-Wallis test	Sexes Combined	*P value*	*K-W statistic*	Dunn's mult. comp. test		*Adjusted P value*	*Mean rank 1*	*Mean rank 2*	*Mean rank diff.*	*n1*	*n2*
			**0.0029**	13.97		*Wt* vs. *Lnx1*$^{-/-}$	0.4433	42.12	54.79	-12.68	30	29
						Wt vs. *Lnx2*$^{-/-}$	**0.0013**	42.12	73.54	-31.42	30	27
						Wt vs. *Lnx1/2*$^{-/-}$	**0.0258**	42.12	64.93	-22.82	30	30
Number of transition	ANOVA summary	Sexes Combined	*P value*	*F-statistic*	Dunnett's mult. comp. test		*Adjusted P value*	*Mean 1*	*Mean 2*	*Mean diff.*	*n1*	*n2*
			**0.0383**	2.897		*Wt* vs. *Lnx1*$^{-/-}$	0.516	14.37	16.28	-1.909	30	29
						Wt vs. *Lnx2*$^{-/-}$	0.9593	14.37	15.04	-0.6704	30	27
						Wt vs. *Lnx1/2*$^{-/-}$	**0.0194**	14.37	18.83	-4.467	30	30

WIRE BEAM BRIDGE

Measure	Test	Sex	P value	Statistic	Post hoc test	Comparison	Adjusted P value	Mean rank 1	Mean rank 2	Mean rank diff.	n1	n2
Latency to access the bridge	Kruskal-Wallis test	Sexes Combined	*P value*	*K-W statistic*	Dunn's mult. comp. test		*Adjusted P value*	*Mean rank 1*	*Mean rank 2*	*Mean rank diff.*	*n1*	*n2*
			**0.0002**	19.94		*Wt* vs. *Lnx1*$^{-/-}$	>0.9999	69.93	71.4	-1.463	30	29
						Wt vs. *Lnx2*$^{-/-}$	**0.0266**	69.93	46.77	23.16	30	26
						Wt vs. *Lnx1/2*$^{-/-}$	**0.0018**	69.93	40.36	29.57	30	29
Latency to cross the bridge	Kruskal-Wallis test	Sexes Combined	*P value*	*K-W statistic*	Dunn's mult. comp. test		*Adjusted P value*	*Mean rank 1*	*Mean rank 2*	*Mean rank diff.*	*n1*	*n2*
			**0.0035**	13.6		*Wt* vs. *Lnx1*$^{-/-}$	>0.9999	67.42	68.03	-0.6178	30	29
						Wt vs. *Lnx2*$^{-/-}$	0.3048	67.42	52.94	14.47	30	26
						Wt vs. *Lnx1/2*$^{-/-}$	**0.0058**	67.42	40.79	26.62	30	29

MARBLE BURYING

Measure	Test	Sex	P value	Statistic	Post hoc test	Comparison	Adjusted P value	Mean rank 1	Mean rank 2	Mean rank diff.	n1	n2
Number of marbles buried	Kruskal-Wallis test	Males	*P value*	*K-W statistic*	Dunn's mult. comp. test		*Adjusted P value*	*Mean rank 1*	*Mean rank 2*	*Mean rank diff.*	*n1*	*n2*
			0.1907	4.755		*Wt* vs. *Lnx1*$^{-/-}$	0.1653	35	22.8	12.2	15	15
						Wt vs. *Lnx2*$^{-/-}$	>0.9999	35	29.7	5.3	15	15
						Wt vs. *Lnx1/2*$^{-/-}$	>0.9999	35	34.5	0.5	15	15
	ANOVA	Females	*P value*	*F-statistic*	Dunnett's mult. comp. test							
			0.2867	1.292		*Wt* vs. *Lnx1*$^{-/-}$	>0.9999	31	33.97	-2.967	14	15
						Wt vs. *Lnx2*$^{-/-}$	>0.9999	31	27.89	3.107	14	14
						Wt vs. *Lnx1/2*$^{-/-}$	0.5692	31	22.79	8.214	14	14

				P value	K-W statistic			Adjusted P value	Mean rank 1	Mean rank 2	Mean rank diff.	n1	n2
**STRESS-INDUCED HYPERTHERMIA**	Change in temperature	Kruskal-Wallis test	Males	0.14	5.477	Dunn's mult. comp. test	*Wt* vs. *Lnx1*$^{-/-}$	0.3735	25.93	35.5	-9.567	15	15
							Wt vs. *Lnx2*$^{-/-}$	>0.9999	25.93	23.57	2.362	15	15
							Wt vs. *Lnx1/2*$^{-/-}$	0.497	25.93	34.57	-8.633	15	15
			Females	0.6618	1.589		*Wt* vs. *Lnx1*$^{-/-}$	>0.9999	30.4	30.36	0.04286	15	14
							Wt vs. *Lnx2*$^{-/-}$	0.795	30.4	23.38	7.025	15	12
							Wt vs. *Lnx1/2*$^{-/-}$	>0.9999	30.4	28.97	1.433	15	15

**NOVEL OBJECT RECOGNITION**	*Day 2*												
				P value	*F-statistic*			*Adjusted P Value*	*Mean 1*	*Mean 2*	*Mean diff.*	*n1*	*n2*
	Interaction time	ANOVA summary	Sexes Combined	0.4595	0.869	Dunnett's mult. comp. test	*Wt* vs. *Lnx1*$^{-/-}$	0.8857	88.32	93.62	-5.302	30	29
							Wt vs. *Lnx2*$^{-/-}$	0.5655	88.32	98.29	-9.971	30	27
							Wt vs. *Lnx1/2*$^{-/-}$	0.2969	88.32	101.8	-13.51	30	31
	Day 3												
				P value	*K-W statistic*			*Adjusted P Value*	*Mean rank 1*	*Mean rank 2*	*Mean rank diff.*		
	Interaction time	Kruskal-Wallis test	Sexes Combined	**<ins>< 0.0001</ins>**	49.73	Dunn's mult. comp. test	*Wt familiar vs. novel*	**<ins>0.0423</ins>**	63.52	107	-43.48	27	27
							Lnx1$^{-/-}$ *familiar vs. novel*	**<ins>0.0091</ins>**	81.5	134.4	-52.92	26	26
							Lnx2$^{-/-}$ *familiar vs. novel*	**<ins>0.0003</ins>**	81.32	152	-70.68	25	25
							Lnx1/2$^{-/-}$ *familiar vs. novel*	**<ins>0.0483</ins>**	102.8	143.3	-40.5	30	30
				P value	*F-statistic*			*Adjusted P Value*	*Mean 1*	*Mean 2*	*Mean diff.*		
	Discrimination index	ANOVA summary	Sexes Combined	0.5817	0.6283	Dunnett's mult. comp. test	*Wt* vs. *Lnx1*$^{-/-}$	0.8608	57.41	59.62	-2.208	28	26
							Wt vs. *Lnx2*$^{-/-}$	0.7911	57.41	60.03	-2.621	28	26
							Wt vs. *Lnx1/2*$^{-/-}$	0.9662	57.41	56.16	1.249	28	30

ULTRASONIC VOCALISATION

Measure	Test	Sex	P value	K-W statistic	Multiple comparison test	Comparison	Adjusted P value	Mean rank 1	Mean rank 2	Mean rank diff.	n1	n2
Number of USVs	Kruskal-Wallis test	Males	0.8482	0.8052	Dunn's mult. comp. test	*Wt* vs. *Lnx1* $^{-/-}$	>0.9999	27.38	31.79	-4.419	16	17
						Wt vs. *Lnx2* $^{-/-}$	>0.9999	27.38	30.57	-3.192	16	15
						Wt vs. *Lnx1/2* $^{-/-}$	>0.9999	27.38	32.75	-5.375	16	12
		Females	0.4985	2.374		*Wt* vs. *Lnx1* $^{-/-}$	0.6173	21.83	28.97	-7.133	15	15
						Wt vs. *Lnx2* $^{-/-}$	0.5777	21.83	29.63	-7.792	15	12
						Wt vs. *Lnx1/2* $^{-/-}$	0.8301	21.83	28.5	-6.667	15	11

Measure	Test	Sex	P value	F-statistic	Multiple comparison test	Comparison	Adjusted P value	Mean 1	Mean 2	Mean diff.	n1	n2
USVs length	ANOVA summary	Males	0.2423	1.435	Dunnett's mult. comp. test	*Wt* vs. *Lnx1* $^{-/-}$	0.7959	0.03568	0.03378	0.00191	16	17
						Wt vs. *Lnx2* $^{-/-}$	0.1694	0.03568	0.03076	0.00492	16	14
						Wt vs. *Lnx1/2* $^{-/-}$	0.2945	0.03568	0.0314	0.004287	16	12
		Females	0.0894	2.3		*Wt* vs. *Lnx1* $^{-/-}$	0.7446	0.03003	0.03242	-0.002386	15	14
						Wt vs. *Lnx2* $^{-/-}$	0.9563	0.03003	0.03126	-0.001231	15	12
						Wt vs. *Lnx1/2* $^{-/-}$	**0.0404**	0.03003	0.03786	-0.007833	15	10
Principal frequency	ANOVA summary	Males	**0.0129**	3.935	Dunnett's mult. comp. test	*Wt* vs. *Lnx1* $^{-/-}$	0.1228	81.48	78.44	3.037	16	17
						Wt vs. *Lnx2* $^{-/-}$	0.5774	81.48	83.19	-1.712	16	14
						Wt vs. *Lnx1/2* $^{-/-}$	0.2804	81.48	78.87	2.607	16	12
		Females	**0.0293**	3.27		*Wt* vs. *Lnx1* $^{-/-}$	0.1183	86.44	82.03	4.416	15	14
						Wt vs. *Lnx2* $^{-/-}$	0.2298	86.44	82.61	3.831	15	12
						Wt vs. *Lnx1/2* $^{-/-}$	**0.0115**	86.44	79.29	7.149	15	10
Delta frequency	ANOVA summary	Males	0.8994	0.195	Dunnett's mult. comp. test	*Wt* vs. *Lnx1* $^{-/-}$	0.9968	21.34	21.05	0.2877	16	17
						Wt vs. *Lnx2* $^{-/-}$	0.8125	21.34	20.02	1.315	16	14
						Wt vs. *Lnx1/2* $^{-/-}$	0.9782	21.34	20.73	0.6084	16	12
		Females	**0.0115**	4.101		*Wt* vs. *Lnx1* $^{-/-}$	0.376	17.08	20.19	-3.115	15	14
						Wt vs. *Lnx2* $^{-/-}$	0.1405	17.08	21.6	-4.527	15	12
						Wt vs. *Lnx1/2* $^{-/-}$	**0.0035**	17.08	25.45	-8.372	15	10

ULTRASONIC VOCALISATION				P value	F-statistic			Adjusted P value	Mean 1	Mean 2	Mean diff.	n1	n2
	Mean power	ANOVA summary	Males	0.1664	1.756	Dunnett's mult. comp. test	*Wt* vs. *Lnx1*$^{-/-}$	0.3814	-85.36	-84.3	-1.067	16	17
							Wt vs. *Lnx2*$^{-/-}$	0.7376	-85.36	-86.05	0.6852	16	14
							Wt vs. *Lnx1/2*$^{-/-}$	0.8577	-85.36	-84.82	-0.5479	16	12
			Females	**0.0003**	7.513		*Wt* vs. *Lnx1*$^{-/-}$	**0.0141**	-87.31	-84.79	-2.515	15	14
							Wt vs. *Lnx2*$^{-/-}$	0.1464	-87.31	-85.58	-1.732	15	12
							Wt vs. *Lnx1/2*$^{-/-}$	**<0.0001**	-87.31	-82.97	-4.341	15	10
				P value	K-W statistic			Adjusted P value	Mean rank 1	Mean rank 2	Mean rank diff.	n1	n2
	Latency	Kruskal-Wallis test	Males	0.9929	0.09055	Dunn's mult. comp. test	*Wt* vs. *Lnx1*$^{-/-}$	>0.9999	29.31	29.5	-0.1875	16	17
							Wt vs. *Lnx2*$^{-/-}$	>0.9999	29.31	30.93	-1.621	16	14
							Wt vs. *Lnx1/2*$^{-/-}$	>0.9999	29.31	30.42	-1.104	16	12
			Females	0.7551	1.191		*Wt* vs. *Lnx1*$^{-/-}$	>0.9999	28.47	27.36	1.11	15	14
							Wt vs. *Lnx2*$^{-/-}$	>0.9999	28.47	23.08	5.383	15	12
							Wt vs. *Lnx1/2*$^{-/-}$	>0.9999	28.47	23.9	4.567	15	10

USV: CALL CLASSIFICATION				P value	K-W statistic			Adjusted P Value	Mean rank 1	Mean rank 2	Mean rank diff.	n1	n2
	Up	Kruskal-Wallis test	Males	0.1068	6.100	Dunn's mult. comp. test	*Wt* vs. *Lnx1*$^{-/-}$	>0.9999	33.69	28.76	4.923	16	17
							Wt vs. *Lnx2*$^{-/-}$	>0.9999	33.69	35.57	-1.884	16	14
							Wt vs. *Lnx1/2*$^{-/-}$	0.1252	33.69	20.33	13.35	16	12
			Females	0.1753	4.953		*Wt* vs. *Lnx1*$^{-/-}$	>0.9999	26.23	25.57	0.6619	15	14
							Wt vs. *Lnx2*$^{-/-}$	0.8196	26.23	32.54	-6.308	15	12
							Wt vs. *Lnx1/2*$^{-/-}$	0.5904	26.23	18.4	7.833	15	10
				P value	K-W statistic			Adjusted P Value	Mean rank 1	Mean rank 2	Mean rank diff.	n1	n2
	Down	Kruskal-Wallis test	Males	**0.0164**	10.270	Dunn's mult. comp. test	*Wt* vs. *Lnx1*$^{-/-}$	0.1194	23.94	36.24	-12.3	16	17
							Wt vs. *Lnx2*$^{-/-}$	>0.9999	23.94	22	1.938	16	14
							Wt vs. *Lnx1/2*$^{-/-}$	0.0766	23.94	38.58	-14.65	16	12
			Females	0.5898	1.917		*Wt* vs. *Lnx1*$^{-/-}$	>0.9999	27.33	29.54	-2.202	15	14
							Wt vs. *Lnx2*$^{-/-}$	>0.9999	27.33	22.54	4.792	15	12
							Wt vs. *Lnx1/2*$^{-/-}$	>0.9999	27.33	23.2	4.133	15	10

ULTRASONIC VOCALISATION: CALL CLASSIFICATION

Call type	Test	Sex	P value	K-W statistic	Post hoc test	Comparison	Adjusted P Value	Mean rank 1	Mean rank 2	Mean rank diff.	n1	n2
Short	Kruskal-Wallis test	Males	0.3349	3.393	Dunn's mult. comp. test	*Wt* vs. *Lnx1*$^{-/-}$	0.3397	23.63	33.09	-9.463	16	17
						Wt vs. *Lnx2*$^{-/-}$	0.3316	23.63	33.64	-10.02	16	14
						Wt vs. *Lnx1/2*$^{-/-}$	>0.9999	23.63	29.88	-6.25	16	12
		Females	0.5755	1.985		*Wt* vs. *Lnx1*$^{-/-}$	>0.9999	29.5	26.39	3.107	15	14
						Wt vs. *Lnx2*$^{-/-}$	>0.9999	29.5	25.29	4.208	15	12
						Wt vs. *Lnx1/2*$^{-/-}$	0.489	29.5	21.05	8.45	15	10
Chevron	Kruskal-Wallis test	Males	**0.0057**	12.570	Dunn's mult. comp. test	*Wt* vs. *Lnx1*$^{-/-}$	**0.0268**	40.94	25.29	15.64	16	17
						Wt vs. *Lnx2*$^{-/-}$	0.5061	40.94	32.29	8.652	16	14
						Wt vs. *Lnx1/2*$^{-/-}$	**0.0031**	40.94	19.42	21.52	16	12
		Females	0.5034	2.348		*Wt* vs. *Lnx1*$^{-/-}$	0.9258	30.77	25.14	5.624	15	14
						Wt vs. *Lnx2*$^{-/-}$	0.4979	30.77	22.79	7.975	15	12
						Wt vs. *Lnx1/2*$^{-/-}$	0.7734	30.77	23.9	6.867	15	10
Complex	Kruskal-Wallis test	Males	**<0.0001**	25.450	Dunn's mult. comp. test	*Wt* vs. *Lnx1*$^{-/-}$	**0.0001**	41.88	17.26	24.61	16	17
						Wt vs. *Lnx2*$^{-/-}$	>0.9999	41.88	40.04	1.839	16	14
						Wt vs. *Lnx1/2*$^{-/-}$	**0.0034**	41.88	20.5	21.38	16	12
		Females	**0.0080**	11.82		*Wt* vs. *Lnx1*$^{-/-}$	**0.0035**	36.63	18.68	17.95	15	14
						Wt vs. *Lnx2*$^{-/-}$	0.0944	36.63	24.25	12.38	15	12
						Wt vs. *Lnx1/2*$^{-/-}$	0.0570	36.63	22.4	14.23	15	10
Multi simple	Kruskal-Wallis test	Males	**0.026**	9.262	Dunn's mult. comp. test	*Wt* vs. *Lnx1*$^{-/-}$	**0.0343**	20.81	35.94	-15.13	16	17
						Wt vs. *Lnx2*$^{-/-}$	0.9832	20.81	26.96	-6.152	16	14
						Wt vs. *Lnx1/2*$^{-/-}$	**0.0347**	20.81	37.38	-16.56	16	12
		Females	**0.0009**	16.44		*Wt* vs. *Lnx1*$^{-/-}$	0.0589	14.93	27.82	-12.89	15	14
						Wt vs. *Lnx2*$^{-/-}$	0.1225	14.93	26.71	-11.78	15	12
						Wt vs. *Lnx1/2*$^{-/-}$	**0.0002**	14.93	39.2	-24.27	15	10
Multi complex	Kruskal-Wallis test	Males	0.6034	1.853	Dunn's mult. comp. test	*Wt* vs. *Lnx1*$^{-/-}$	>0.9999	32.47	30.35	2.116	16	17
						Wt vs. *Lnx2*$^{-/-}$	>0.9999	32.47	31.68	0.7902	16	14
						Wt vs. *Lnx1/2*$^{-/-}$	0.6168	32.47	24.25	8.219	16	12
		Females	0.6567	1.612		*Wt* vs. *Lnx1*$^{-/-}$	0.9686	22.17	27.57	-5.405	15	14
						Wt vs. *Lnx2*$^{-/-}$	0.7348	22.17	28.79	-6.625	15	12
						Wt vs. *Lnx1/2*$^{-/-}$	>0.9999	22.17	26.2	-4.033	15	10

				P value#				*Adjusted P Value*	*Mean 1*	*Mean 2*	*Mean diff.*	*n1*	*n2*
**BODY MASS**		Two-way ANOVA with repeated measures performed on rank transformed data	Males	**<0.0001**	Dunnett's multiple comparisons test	week 1	*Wt* vs. *Lnx1*$^{-/-}$	**<0.0001**	4.464	3.01	1.454	28	30
							Wt vs. *Lnx2*$^{-/-}$	**0.0012**	4.464	3.917	0.5469	28	23
							Wt vs. *Lnx1/2*$^{-/-}$	**<0.0001**	4.464	3.217	1.247	28	29
						week 2	*Wt* vs. *Lnx1*$^{-/-}$	**<0.0001**	7.711	6.027	1.684	28	30
							Wt vs. *Lnx2*$^{-/-}$	0.4052	7.711	7.365	0.3455	28	23
							Wt vs. *Lnx1/2*$^{-/-}$	**<0.0001**	7.711	6.186	1.525	28	29
						week 3	*Wt* vs. *Lnx1*$^{-/-}$	**<0.0001**	9.468	7.403	2.065	28	30
							Wt vs. *Lnx2*$^{-/-}$	0.9911	9.468	9.383	0.08525	28	23
							Wt vs. *Lnx1/2*$^{-/-}$	**<0.0001**	9.468	7.469	1.999	28	29
						week 4	*Wt* vs. *Lnx1*$^{-/-}$	**<0.0001**	15.49	13	2.486	28	30
							Wt vs. *Lnx2*$^{-/-}$	0.97	15.49	15.68	-0.1969	28	23
							Wt vs. *Lnx1/2*$^{-/-}$	**<0.0001**	15.49	13.03	2.451	28	29
						week 5	*Wt* vs. *Lnx1*$^{-/-}$	**<0.0001**	20.5	18.24	2.256	28	30
							Wt vs. *Lnx2*$^{-/-}$	>0.9999	20.5	20.52	-0.02531	28	23
							Wt vs. *Lnx1/2*$^{-/-}$	**<0.0001**	20.5	18.22	2.279	28	29
						week 7	*Wt* vs. *Lnx1*$^{-/-}$	**0.0009**	24.23	22.21	2.022	28	30
							Wt vs. *Lnx2*$^{-/-}$	>0.9999	24.23	24.22	0.01118	28	23
							Wt vs. *Lnx1/2*$^{-/-}$	**0.0004**	24.23	21.98	2.246	28	29
						week 8	*Wt* vs. *Lnx1*$^{-/-}$	**0.0008**	25.65	23.5	2.147	28	30
							Wt vs. *Lnx2*$^{-/-}$	0.9156	25.65	25.3	0.3457	28	23
							Wt vs. *Lnx1/2*$^{-/-}$	**0.0001**	25.65	23.26	2.388	28	29

				P value [#]				*Adjusted P Value*	*Mean 1*	*Mean 2*	*Mean diff.*	*n1*	*n2*
**BODY MASS**		Two-way ANOVA with repeated measures performed on rank transformed data	Females	**<0.0001**	Dunnett's multiple comparisons test	week 1	*Wt* vs. *Lnx1* $^{-/-}$	**<0.0001**	4.135	2.944	1.19	26	27
							Wt vs. *Lnx2* $^{-/-}$	**0.0192**	4.135	3.776	0.3586	26	25
							Wt vs. *Lnx1/2* $^{-/-}$	**<0.0001**	4.135	3.281	0.8538	26	26
						week 2	*Wt* vs. *Lnx1* $^{-/-}$	**<0.0001**	7.342	5.889	1.453		
							Wt vs. *Lnx2* $^{-/-}$	0.7662	7.342	7.148	0.1943		
							Wt vs. *Lnx1/2* $^{-/-}$	**<0.0001**	7.342	6.065	1.277		
						week 3	*Wt* vs. *Lnx1* $^{-/-}$	**<0.0001**	9.015	7.041	1.975		
							Wt vs. *Lnx2* $^{-/-}$	0.9305	9.015	8.872	0.1434		
							Wt vs. *Lnx1/2* $^{-/-}$	**<0.0001**	9.015	7.308	1.708		
						week 4	*Wt* vs. *Lnx1* $^{-/-}$	**<0.0001**	12.95	10.76	2.191		
							Wt vs. *Lnx2* $^{-/-}$	0.436	12.95	12.45	0.5058		
							Wt vs. *Lnx1/2* $^{-/-}$	**<0.0001**	12.95	11.37	1.585		
						week 5	*Wt* vs. *Lnx1* $^{-/-}$	**0.0404**	14.95	13.82	1.132		
							Wt vs. *Lnx2* $^{-/-}$	0.9487	14.95	15.12	-0.1662		
							Wt vs. *Lnx1/2* $^{-/-}$	**0.0006**	14.95	13.55	1.408		
						week 7	*Wt* vs. *Lnx1* $^{-/-}$	**0.0002**	17.7	15.78	1.918		
							Wt vs. *Lnx2* $^{-/-}$	0.4127	17.7	18.22	-0.5278		
							Wt vs. *Lnx1/2* $^{-/-}$	**0.0004**	17.7	16	1.7		
						week 8	*Wt* vs. *Lnx1* $^{-/-}$	**0.0002**	18.3	16.29	2.007		
							Wt vs. *Lnx2* $^{-/-}$	0.9977	18.3	18.36	-0.064		
							Wt vs. *Lnx1/2* $^{-/-}$	**0.0003**	18.3	16.45	1.85		

[#] *genotype as source of variation*
